# The Population and Evolutionary Dynamics of Phage and Bacteria with CRISPR–Mediated Immunity

**DOI:** 10.1371/journal.pgen.1003312

**Published:** 2013-03-14

**Authors:** Bruce R. Levin, Sylvain Moineau, Mary Bushman, Rodolphe Barrangou

**Affiliations:** 1 Department of Biology, Emory University, Atlanta, Georgia, United States of America; 2 Département de Biochimie, Microbiologie et Bio-Informatique, Faculté des Sciences et de Génie, Groupe de Recherche en Écologie Buccale, Faculté de Médecine Dentaire, Félix d'Hérelle Reference Center for Bacterial Viruses, Université Laval, Laval, Québec, Canada; 3 Molecules to Mankind Program and Graduate Program in Population Biology, Ecology and Evolution (PBEE), Laney Graduate School, Emory University, Atlanta, Georgia, United States of America; 4 DuPont Nutrition and Health, Madison, Wisconsin, United States of America; Uppsala University, Sweden

## Abstract

Clustered Regularly Interspaced Short Palindromic Repeats (CRISPR), together with associated genes (*cas*), form the CRISPR–*cas* adaptive immune system, which can provide resistance to viruses and plasmids in bacteria and archaea. Here, we use mathematical models, population dynamic experiments, and DNA sequence analyses to investigate the host–phage interactions in a model CRISPR–*cas* system, *Streptococcus thermophilus* DGCC7710 and its virulent phage 2972. At the molecular level, the bacteriophage-immune mutant bacteria (BIMs) and CRISPR–escape mutant phage (CEMs) obtained in this study are consistent with those anticipated from an iterative model of this adaptive immune system: resistance by the addition of novel spacers and phage evasion of resistance by mutation in matching sequences or flanking motifs. While CRISPR BIMs were readily isolated and CEMs generated at high rates (frequencies in excess of 10^−6^), our population studies indicate that there is more to the dynamics of phage–host interactions and the establishment of a BIM–CEM arms race than predicted from existing assumptions about phage infection and CRISPR*–cas* immunity. Among the unanticipated observations are: (i) the invasion of phage into populations of BIMs resistant by the acquisition of one (but not two) spacers, (ii) the survival of sensitive bacteria despite the presence of high densities of phage, and (iii) the maintenance of phage-limited communities due to the failure of even two-spacer BIMs to become established in populations with wild-type bacteria and phage. We attribute (i) to incomplete resistance of single-spacer BIMs. Based on the results of additional modeling and experiments, we postulate that (ii) and (iii) can be attributed to the phage infection-associated production of enzymes or other compounds that induce phenotypic phage resistance in sensitive bacteria and kill resistant BIMs. We present evidence in support of these hypotheses and discuss the implications of these results for the ecology and (co)evolution of bacteria and phage.

## Author Summary

The evidence that the CRISPR regions of the genomes of archaea and bacteria play a role in the ecology and (co)evolution of these microbes and their viruses is overwhelming: (i) the spacers (variable sequences of 26–72 bp of DNA between the repeats of this region) of these prokaryotes are homologous to the DNA of viruses in their communities; (ii) experimentally, the acquisition and incorporation of spacers of viral DNA can protect these organisms from subsequent infection by these viruses; (iii) experimentally, viruses evade this immunity by mutation in homologous protospacers or protospacer-adjacent motifs (PAMs). Not so clear are the nature and magnitude of the role CRISPR plays in this ecology and evolution. Here, we use mathematical models, experiments with *Streptococcus thermophilus* and the phage 2972, and DNA sequence analyses to explore the contribution of CRISPR*–cas* immunity to the ecology and (co)evolution of bacteria and their viruses. The results of this study suggest that the contribution of CRISPR to the ecology of bacteria and phage is more modest and limited, and the conditions for a CRISPR–mediated coevolutionary arms race between these organisms more restrictive, than anticipated from models based on the canonical view of phage infection and CRISPR*–cas* immunity.

## Introduction

The experimental demonstrations, in 2007 and 2008, that the Clustered Regularly Interspaced Short Palindromic Repeats that abound in the genomes of the vast majority of archaea and nearly half of bacteria can serve as part of an adaptive immune system (CRISPR–*cas*) that protects these prokaryotes against lytic phage [1] and conjugative plasmids [2] raised many intriguing questions and stimulated a great deal of research. Between 2007 and 2012, according to PubMed, the number of articles with the acronym CRISPR in their title and/or body has been doubling every 20 months. Understandably, the majority of these articles are reports or reviews of the genetic, biochemical, and molecular mechanisms by which (i) the CRISPR–*cas* system acquires the 26–72 base pair sequences (spacers) of DNA from infecting phage, generating Bacteriophage Insensitive Mutants (BIMs), or from invading plasmids, forming Plasmid Interfering Mutants (PIMs); (ii) the small RNAs coded for by these spacers abort subsequent infections by phage and plasmids with DNA sequences corresponding to those in these spacers (protospacers) [3], [4], [5], [6]; (iii) the phage can evade this resistance and generate CRISPR Escape Mutants (CEMs) by modifying the DNA sequences of protospacers or protospacer adjacent motifs (PAM) [3], [7], [8], [9]; (iv) the Cas proteins participate in these processes [1], [8], [9]; and (v) the existence, distribution and genetic diversity of CRISPR–*cas* systems in the archaea and bacteria came about [10], [11], [12]

Some of this research has been devoted to the practical applications of CRISPR and the CRISPR–*cas* system. Long before the immunological role of CRISPR in these microorganisms was recognized, differences in the number of these palindromic repeats had been used as markers for studies of the genetic (molecular) epidemiology and forensics of pathogenic bacteria [13], [14], [15], [16]. Isolates that would be classified as identical by standard typing procedures, like Serotyping, Multi-Locus Sequence Typing, and Pulse Field Gel Electrophoresis, can differ in their CRISPR regions, particularly in their spacer sequences [17], [18]. Genes, and clusters of genes, borne on plasmids, phage, or chromosomal genes, and acquired by horizontal transfer, code for the virulence and antibiotic resistance of many pathogenic bacteria. And, as one might anticipate, strains of at least some species of pathogenic bacteria that lack functional CRISPR–*cas* systems are more likely to be virulent and resistant to antibiotics than those that bear these systems [19]. A number of biotechnological and industrial processes rely on the use of bacteria that are subject to infection by phage, and therefore risk disruption of production, low-quality products and economic loss. Can CRISPR–*cas* systems be harnessed to counter antibiotic resistance and virulence of pathogenic bacteria and/or prevent phage contamination of industrially important bacteria (8)?

Central to understanding these practical implications and potential applications of CRISPR–*cas* systems are the population and evolutionary dynamics of bacteria bearing these adaptive immune systems and those of the phage and plasmids that infect them. Prior to the experimental demonstration that CRISPR is part of an adaptive immune system, and motivating those experiments, were observations that the spacer sequences in the CRISPR regions of bacteria and archaea from natural communities were identical to sequences in the genomes of viruses in their communities [15], [20], [21]. These and more recent studies of bacteria and archaea and their viruses [10], [11], [12], [22], and metagenomic studies of microbiomes [23], provided compelling support for the hypothesis that CRISPR–*cas* systems play an active role in the population dynamics and co-evolution of these organisms in natural communities. However, the inferences one can draw from these investigations are limited. They provide little of the necessary quantitative information about the nature and contribution of CRISPR–*cas* to these dynamics and co-evolution and its consequential role in determining the structure of their communities.

Studies using mathematical models and computer simulations suggest that bacteria with CRISPR–*cas* systems can invade communities of phage and bacteria without this immune system and can also prevent phage from invading their communities [24]. Modeling studies also suggest that, in theory, a CRISPR–*cas*-mediated BIM-CEM co-evolutionary arms race can maintain considerable diversity in the interacting populations of bacteria and phage [25] and account for spacer changes in the CRISPR elements in the metagenomic structure of natural communities of archaea and bacteria and their phage [26]. But, as compelling as these mathematical models and computer simulation studies may be, their limitations are also apparent. The models used are based on simplifying assumptions about the phage infection process and CRISPR–*cas* adaptive immunity that have not been formally tested in the systems under study. The computer simulations and other numerical analyses of the properties of these models use parameter values for which there are few independent estimates.

In this study we use mathematical models, computer simulations, parameter estimation, and population dynamic and other microbiological experiments with the Gram-positive bacteria *Streptococcus thermophilus* and its virulent phage 2972 (*Siphoviridae* family) to explore the nature and contribution of CRISPR–*cas* adaptive immunity to the population and evolutionary dynamics of bacteria and phage. The results of these experiments and the molecular characterization of the bacteria and phage employed demonstrate that the qualitative Lamarckian (iterative acquisition of spacers) [27] and Darwinian (mutation in protospacers) genetic conditions for a CRISPR–mediated arms race are met with this phage-host system. BIMs are readily formed and CEMs capable of growing on these BIMs are generated at high rates. On the other hand, our experimental results and theoretical analyses indicate that even at a qualitative level, models based on existing assumptions about phage infection and CRISPR–mediated resistance cannot account for the population and evolutionary dynamics of the interactions between *S. thermophilus* and phage 2972. Findings contrary to what is anticipated from the analysis of the properties of these models include: (i) the invasion of phage into populations of BIMs resistant due to the acquisition of one (but not two) spacers; (ii) the survival of substantial numbers of sensitive bacteria despite the presence of high densities of phage; and (iii) the maintenance of phage-limited communities due to the failure of even two-spacer BIMs to become established in populations with wild type bacteria and phage. We postulate that the latter two results can be attributed to the phage infection-associated production of enzymes or other compounds that kill resistant BIMs and generate phenotypically phage-resistant “persister” populations of sensitive bacteria. By modifying the model to account for these chemical processes we can explain the observed dynamics. The predictions of this extended model are supported by longer-term population dynamic experiments.

## Results

### Theoretical framework

#### Overview

We open this report with a mathematical model that is based on the classical view of the process of infection of bacteria with lytic phage [28] and the current perspective on CRISPR–*cas*-mediated immunity to phage infection [3]. The roles of this model and the computer simulations used to analyze its properties are to (i) evaluate the contributions of the major parameters governing the dynamics of phage infection of bacteria with CRISPR–*cas* immune systems; (ii) generate testable hypotheses about these dynamics; (iii) guide the design of the experiments testing these hypotheses; and (iv) interpret the results of these experiments. This opening model is in essence a quantitative straw person. It is not intended to provide quantitatively precise descriptions of these dynamics, but rather to identify major qualitative deviations between the results of our experiments and a theory based on the canonical view of phage infection and CRISPR–*cas* immunity. For our analysis of the properties of this model, we use semi-stochastic simulations employing parameter values estimated experimentally in cultures of *S. thermophilus* strain DGCC7710 and phage 2972.

#### The model

**Figure 1:**
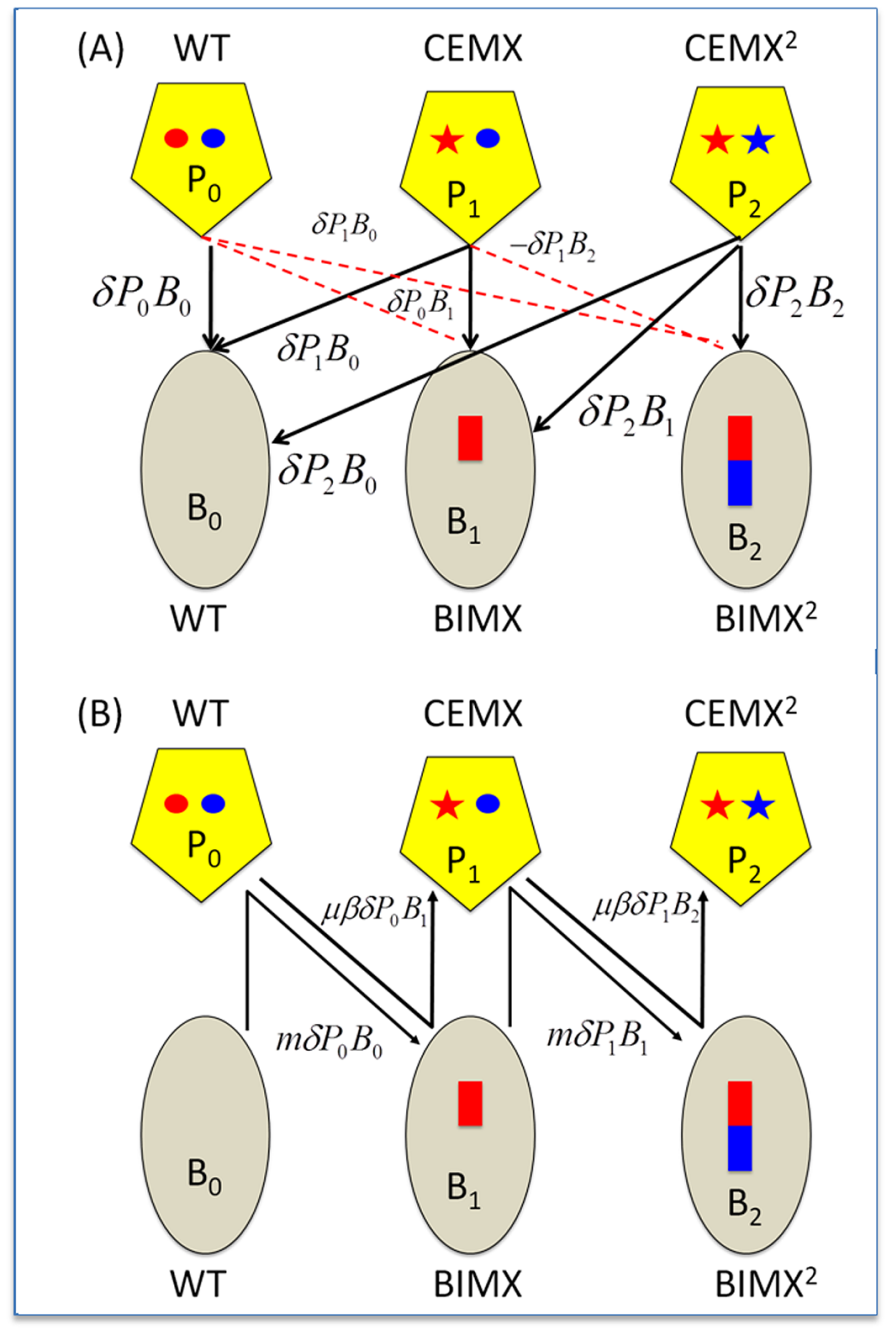
CRISPR–mediated arms race. Ellipses - bacteria; Pentagons - phage; red and blue rectangles - acquired spacers; red and blue circles, regions of the phage genome corresponding to the acquired spacers (protospacers); stars, mutated protospacers generating CEMs. WT, Wild type bacteria and phage; BIMX and CEMX, bacteria with spacers for acquired resistance to WT phage and the first-order CRISPR Escape mutants (CEMX), respectively; BIMX^2^ and CEMX^2^, bacteria with spacers for acquired resistance to CEMX and the second-order CRISPR–escape mutants, respectively. Panel A: Infection and phage replication relationships. Solid black lines, phage adsorption and replication; broken red lines - phage adsorption and loss. Panel B: Changes in state. BIMX are produced by WT phage infecting WT cells and BIMX^2^ are produced by CEMX infecting BIMX. CEMX are produced by P_0_ infecting and replicating on B_0_ and CEMX^2^ are produced by CEMX infecting and replicating on BIMX.

The bacteria and phage are of three possible orders, with densities and designations, respectively, *B_0_*, *B_1_*, and *B_2_*, as well as *P_0_*, *P_1_*, and *P_2_* (Figure 1). Phage are able to adsorb to bacteria of all orders, but are only able to replicate on bacteria of states with the same or lower index numbers (earlier states). For example, phage P_1_ can adsorb to and replicate on B_0_ and B_1_ and adsorb to but not replicate on B_2_. Phage that adsorb to bacteria on which they cannot replicate are lost from their population. Phage resistance (the generation of BIMs) is acquired by the addition of DNA sequences (spacers) into the CRISPR locus that correspond to sequences borne by the infecting phage (protospacers). Although we realize that there are at least two active CRISPR–*cas* systems in this *S. thermophilus* strain, for simplicity, we assume there is a unique functional CRISPR–*cas* system. Spacers accumulate without loss of earlier spacers. By mutation in a phage protospacer corresponding to a host spacer, phage are able to replicate on bacteria with that spacer. These CEMs accumulate protospacer (or PAM) mutations without loss of those acquired earlier and thereby can replicate on bacteria with spacers providing resistance to all earlier incarnations of that phage. For example a phage P_1_ with protospacer mutation that enables it to replicate on bacteria B_1_ can also replicate on B_0_. For simplicity and tractability, we assume that there is a single bacterial and phage genotype within each order as defined by the number of spacers. In reality BIMs may be generated by the acquisition of many different spacers. We consider the implications of this simplifying assumption in the Discussion.

The rate of growth of the bacteria is a monotonic increasing function of the concentration of a limiting resource *r* (µg/ml), 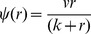, where *v* (per hour) is the maximum growth rate and *k* (µg) is the concentration of the resource where the growth rate is half its maximum value (*v*/2) [29]. Resources are taken up at a rate proportional to the densities of the bacteria, their growth rates, and a conversion efficiency parameter *e* (µg) which is the amount of resource needed to produce a new cell [30].

The phage adsorb to bacteria of all states with a rate parameter δ (ml per cell per phage per hour) and produce β phage per infection (burst size). As in [24], [25], [31], we assume phage infection is a mass action process that occurs at a rate proportional to the product of the densities of the populations of bacteria and phage and the rate constant, δ. Spacers are incorporated only from phage capable of replicating on bacteria of a given state. The parameter *m* is the probability that a bacterium infected by a phage that can replicate on it will add a spacer for immunity to that phage. CEM phage of state *P*_1_ are produced by *P_o_* during the course of replication on *B_0_* with a probability μ per phage per infection. To account for the latent period of length λ hours, we assume that infected bacteria enter a fated state, *M_ij_*, for bacteria of state *i* infected with phage of state *j*. At time *t*, a fraction (1-*m*) of the cells infected λ hours earlier (at time *t*-λ) burst and produce β-1 particles. The (*1-m*) is to account for the fraction of infected B_0_ and B_1_ cells forming BIMs (*B_1_* and *B_2_*, respectively); the β-1 new phage particles accounts for the loss of the infecting phage. CEMs of a given order are formed by mutation occurring during infections of sensitive bacteria by phage of the previous order. For example, phage of state *P_1_* are produced as a fraction μ of the phage particles generated upon burst of B_0_ cells infected with P_0_. With these definitions and assumptions, the rates of change in the concentration of the resource and densities of phage are given by:
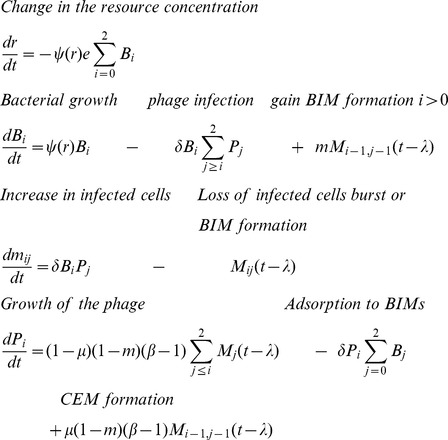



#### Computer simulations

In our numerical solutions to these equations - computer simulations - the acquisition of spacers and CEM mutations are random processes. We use the Euler method for solving the differential equations and a Monte Carlo protocol for the generation of BIMs and CEMs. In any time interval *Δt*, the probability that a BIM is formed is proportional to the number of sensitive cells infected with phage during that time interval. For example, in a given interval *Δt*, the probability that a *P_1_* infecting a *B_1_* will produce a single *B_2_* is approximately *mM_1_(t-λ)Δt* where *t* is time in hours. If a random number *x* (0<*x*<1) from a uniform distribution is less than this probability, a single *B_2_* will enter the *B_2_* population. In any time interval, the probability that a sensitive cell infected with phage will produce a CEM is proportional to the number of phage. For example, the probability that a *P_1_* CEM will be produced by a *P_0_* infecting a *B_0_* in an interval *Δt* is approximately *μM_0_(t-λ)(β-1)Δt*. If a random number *x* is less than this product, a single phage particle will enter the *P_1_* population and be removed from the *P_0_* population. The interval size *Δt* is chosen so that at any time these probabilities are less than 1.0, as they should be. These simulations were programmed in Berkeley Madonna. The program used for the analysis of the properties of this model can be obtained at www.eclf.net/programs.

### Parameter values and short-term population dynamics

#### Bacterial growth

The maximum growth rate of the wild type *S. thermophilus* strain DGCC7710 in LM17 medium (the parameter *v*) was estimated as the regression coefficient of the changes in the natural log of the optical density (OD 600 nm) of growing culture: 1.37±0.27 (95% confidence interval). Since the bacteria were grown in broth rather than a medium with a defined limiting resource, we cannot directly estimate the Monod constant (*k*) or the conversion efficiency (*e*). For the former, we arbitrarily assume a value of 1 µg/ml. Although we cannot directly estimate the parameter *e*, for our simulations we use values of *e* and a maximum resource concentration *R* that, along with the estimated *v* and assumed *k*, provide a reasonable fit to the population growth and saturation (stationary phase density) of wild type *S. thermophilus*.

#### Phage infection

The latent period λ and burst size β were estimated from one-step growth experiments [28]. A late-log culture of wild-type *S. thermophilus* was mixed with an equal volume of LM17Ca for a total volume of 0.9 ml and incubated at 42°C for 10 minutes, at which time 0.1 ml of a LM17Ca-diluted wild-type lysate of 2972 was added. The cell concentration was approximately 2×10^8^ per ml while the phage titer was 10^6^ phage per ml. The culture was incubated for 15 minutes at 42°C and then serially diluted in LM17Ca broth for cell densities and phage titers of approximately (a) 2×10^5^ and 10^3^, (b) 2×10^4^ and 10^2^, and (c) 2×10^3^ and 10^1^. At periodic intervals, 100 µl samples from (a), (b), and (c) were each incubated with 1 ml of a late-log culture of wild type *S. thermophilus* and the number of phage particles was estimated from soft LM17Ca agar lawns. Based on this protocol, the latent period ended between 25 and 30 minutes, thus λ is approximately 0.4 hour. Previous studies [7], [32] estimated the latent period of phage 2972 between 34 to 40 minutes. For the burst size, we use the difference between the mean estimated phage densities in the time intervals 15–25 minutes (before the burst) and 40–60 minutes (after the burst). β was estimated to be approximately 80 particles per infected cell. A previous study estimated the burst size of phage 2972 at 190±33 new virions per infected cell [7]. Presumably these discrepancies can be attributed to differences in the growth conditions, including media.

**Figure 2:**
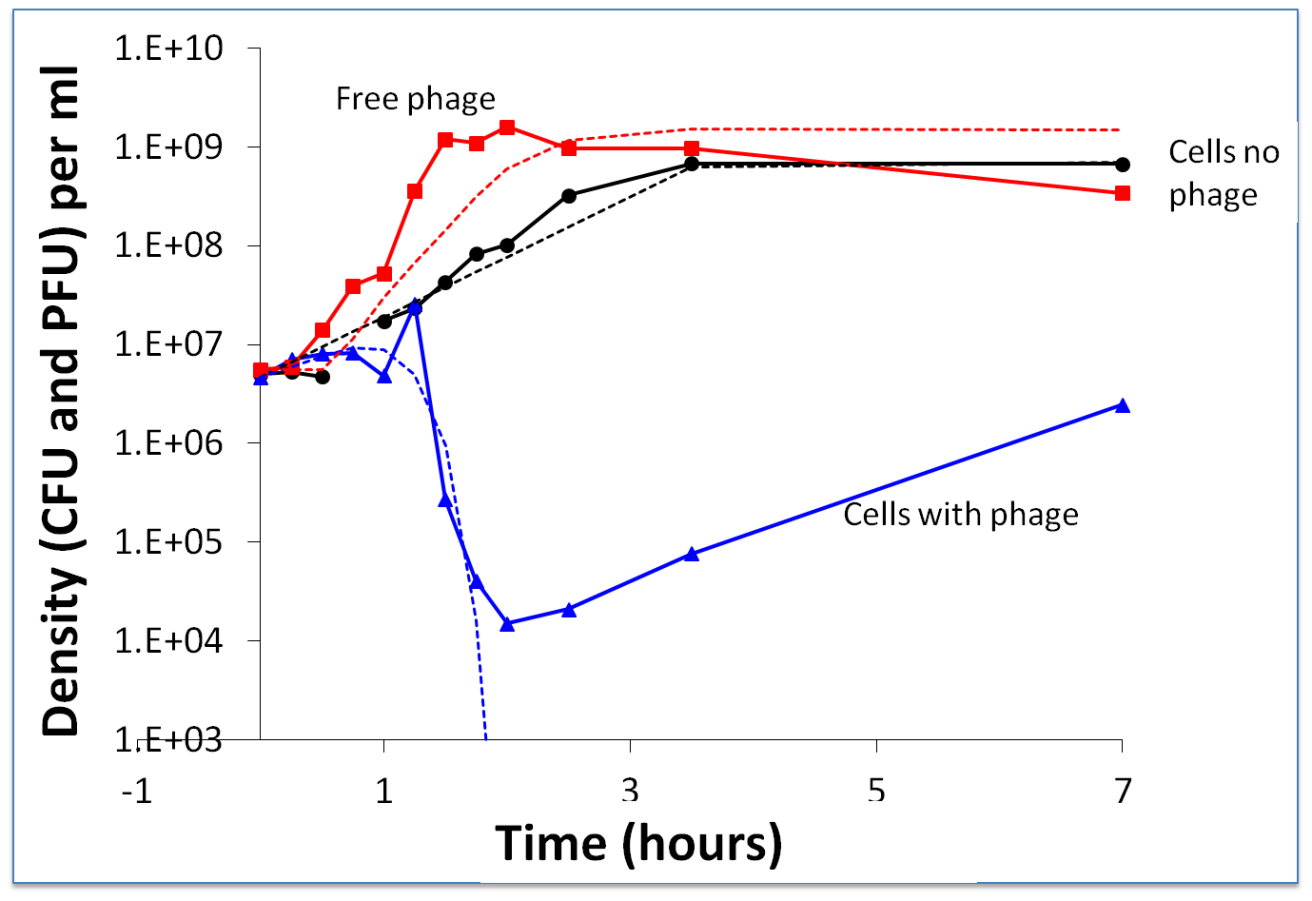
Short-term bacterial and phage growth dynamics in the absence of immunity: change in the density of WT bacteria and WT phage. The broken lines are the densities predicted by the above model with the following parameters: v = 1.4 hr^−1^ per hour, e = 5×10^−7^ µg per cell, R (initial resource concentration) = 350 µg/ml, δ = 8×10^−8^ ml per cell per phage per hour, β = 80 phage particles per infected cell and, λ = 0.4 hours. At 24 hours, the estimated densities of bacteria and phage in these cultures were, respectively, 5×10^8^ cells per ml for the control and 3.2×10^5^ CFU and 6.2×10^7^ PFU for the bacteria and phage in the mixed culture.

There are a variety of ways to obtain independent estimates of the adsorption rate constant δ, but it is usually through the decline in the titers of free phages in bacterial cultures [28]. These methods require separating plaque-forming units (which include infected cells) from free phages. While we tried various protocols, the results were variable, presumably due to the production of exopolysaccharides by the strain DGCC7710 [33], [34]. Moreover, since relatively high densities of cells were required for these estimates (which in theory could be made with BIMs as well as sensitive cells), the physiological state of these bacteria may be different from that of the rapidly growing cultures. Thus, instead of independently estimating this parameter, we used the value of δ that visually provides a good fit for this model for short-term bacterial growth and phage replication experiments with wild-type phage and bacteria. For this fit, we use the above estimates of maximum growth rate (*v*), latent period, and burst size. In this way, δ is the only fitted phage infection parameter (Figure 2).

While the observed and predicted dynamics of the decline in the density of bacteria and the increase in phage titer during the first two hours were similar, from the perspective of the longer-term population dynamics, the most significant difference between the observed and predicted dynamics was the re-ascent and persistence of the bacterial population. In accordance with the model, all of the bacteria should have been killed after two hours of exposure to the phage. The results of the spot tests, made with WT phage and cultures derived from single bacterial colonies recovered from the above at 7–9 and 24–26 hours, showed that these bacteria were still sensitive to the WT phage (more than 20 independent colonies from separate experiments). Stated another way, there was no evidence for BIMs evolving and ascending to dominance in these cultures. This qualitative deviation from what was anticipated under the canonical perspective of the dynamics of lytic phage infection and bacteria with CRISPR–*cas* immunity set the stage for experiments described in the following.

#### Rate of BIM formation (spacer acquisition)

The failure of BIMs to ascend in the experiment depicted in Figure 2 could be due to the rate of BIM formation being too low for these resistant cells to be present in the WT population. Our results suggest this is not the case. BIMs can be readily isolated from lawns initiated with mixtures of 10^8^ phage and 10^7^ cells, and as we demonstrate below. Even when BIMs resistant to the phage are present, they do not ascend in populations dominated by bacteria sensitive to the phage. The exact rate at which BIMs are generated is not clear from our results.

On first consideration, it would seem straightforward to estimate the probability that an infection of a sensitive bacterium with a virulent phage would result in immunity (the acquisition of a spacer) rather than a lytic infection (this probability is the parameter *m* in the above model). Thus, by incubating a mixture of WT cells and WT phage for a defined period, plating the mixture and counting the number of colonies, from the estimated P_0_, B_0_, and δ, it should be possible to estimate *m*. When we did this experiment, the number of surviving colonies varied with the numbers of cells and phage plated, but not in a way anticipated from this model if all of the BIMs were formed in liquid. For example, there were more surviving (resistant) colonies when lower densities of the WT phage and bacteria were plated. We interpret the results of these experiments and some of those that follow as support for the hypothesis that processes other than those considered in the above model affect the rate of BIM formation. In particular, the generation and ascent of BIMs depends on the experimental conditions in ways that we consider below.

#### Frequency of CEM production

In our model, we assume that CEM phage capable of growing on BIMs are produced upon burst of an infected bacterium. While, in our experiments, we did not estimate the rate at which these CEMs are generated, we estimated their frequencies in lysates. Estimating mutation rates, rather than frequencies, in phage requires more information about the way these viruses replicate in infected cells than we currently have for phage 2972 and its *S. thermophilus* host. To estimate the frequency of CEMs capable of replicating on the first-order BIMs, we prepared independent single-plaque lysates of WT phage and estimated the number of particles per ml that formed plaques on 10 different BIMs (BIM1 through BIM10). These 10 BIMs are different from each other based on their newly acquired spacers (see below). With the exception of one WT lysate and the lawns of BIM7 and BIM8, the relative frequency of first-order CEM phage in WT phage lysates was between 5×10^−7^ and 5×10^−5^ (Figure S1). We also obtained single estimates of the frequencies of second-order CEMs from the ratio of the numbers of plaques formed by first-order CEMs on lawns of second-order BIMs and WT lawns, respectively. In a similar experiment we did with the five second-order BIMs and their corresponding first-order CEM phage, the frequency of second-order CEMs also ranged from 5×10^−7^ to 4×10^−6^ (Figure S1).

### Molecular characterization of the BIMs and CEMs

**Figure 3:**
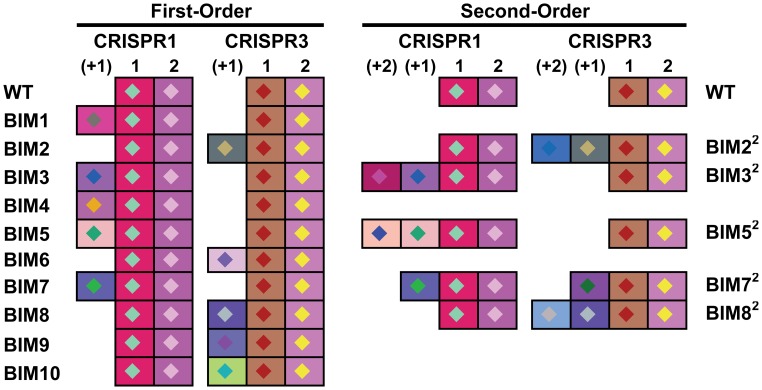
Graphical representation of spacers across the two CRISPR loci for *S. thermophilus* BIMs. Repeats are not included; only spacers are represented. Each spacer is represented by a combination of one select character in a particular color, on a particular background color, as previously described [36]. The color combination allows unique representation of a particular spacer. Similar color schemes (combination of character color and background color) represent identical spacers, whereas different color combinations represent distinguishable spacers.

Genetic analysis of the two active CRISPR loci of ten first-order BIMs revealed that they each had acquired a novel spacer in either the CRISPR1 or CRISPR3 locus. Five BIMs acquired one unique novel spacer in the CRISPR1 locus and 5 BIMs acquired one unique novel spacer in the CRISPR3 locus (Figure 3). Similar analysis of the five second-order BIMs revealed that they each had also acquired an additional novel spacer in the CRISPR1 or CRISPR3 locus. Two of these second-order BIMs acquired one additional unique novel spacer in the CRISPR1 locus, indicating iterative addition into the CRISPR1 locus (BIM3^2^ and BIM5^2^ in Figure 3). Likewise, three second-order BIMs acquired novel spacers in the CRISPR3 locus, including two variants with iterative build-up of the CRISPR3 locus (BIM2^2^ and BIM8^2^ in Figure 3). Interestingly, BIM7^2^ acquired a CRISPR1 spacer to become a first-order CEM and a CRISPR3 spacer to become a second-order CEM, illustrating the duality of active CRISPR loci in *Streptococcus thermophilus*.

Sequence analysis of all the CRISPR1 and CRISPR3 spacers acquired in the first and second rounds of phage challenges indicated perfect matches to the WT genome of phage 2972 used in the challenge (Table S1). Further, all new CRISPR1 and CRISPR3 spacers were associated with protospacer-adjacent motifs (PAMs): NNAGAAW and NGGNG, respectively (Table S1). These findings are consistent with previous results indicating that spacer sequences are directly derived from phage DNA and are associated with PAMs [7], [8], [35], [36].

**Figure 4:**
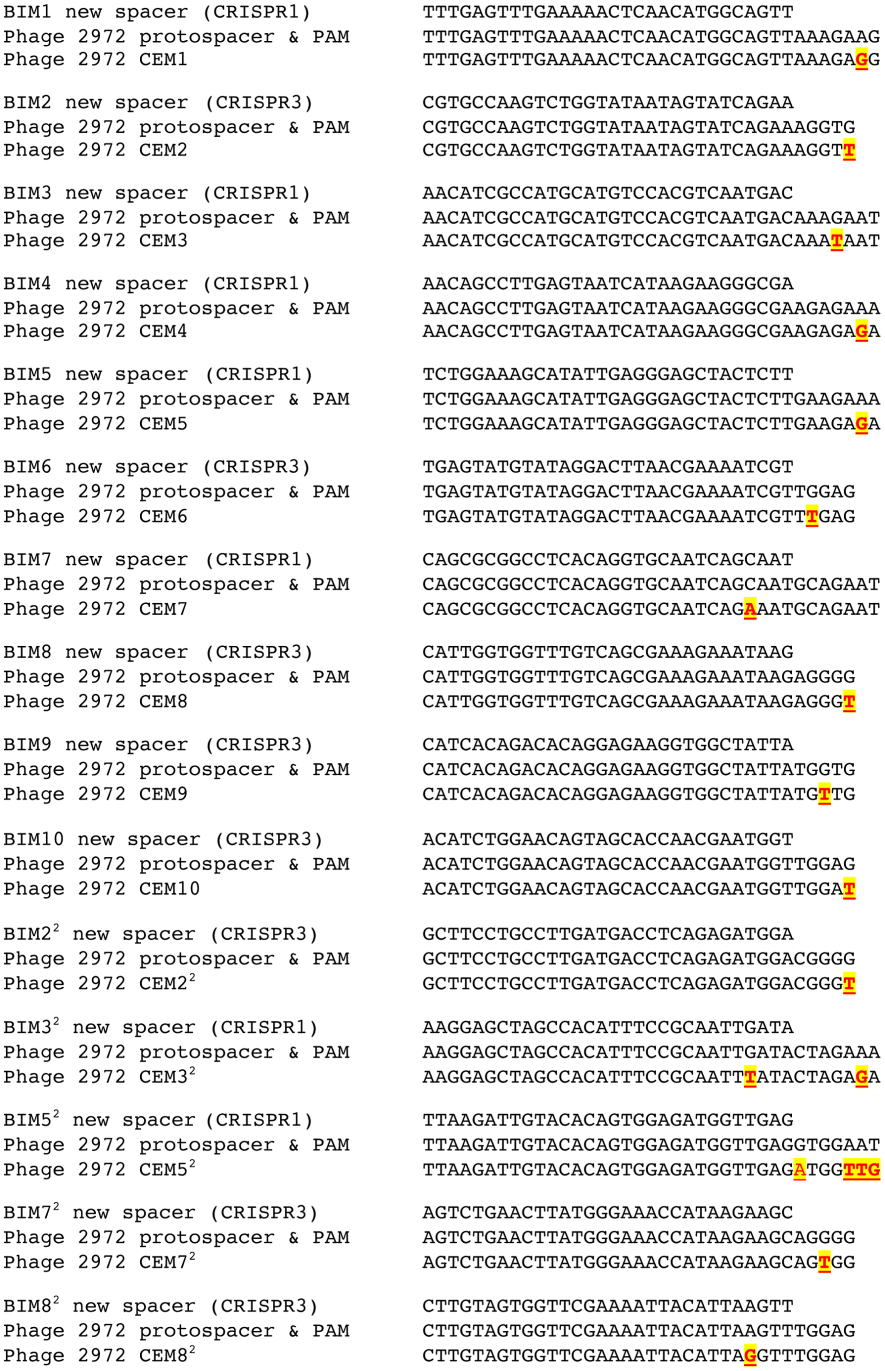
Nucleotide sequences in wild-type and mutant phages that correspond to the newly acquired spacers by the *S. thermophilus* BIM strains. Each mutant is highlighted in red and yellow, bold and underlined.

The results of our sequence analysis of the CEM protospacer and PAM regions are fully consistent with previous findings that single mutations in the matching protospacer or PAM are sufficient to circumvent CRISPR–mediated immunity (Figure 4). Of the 10 CEMs that evaded the immunity of the first-order BIMs, one had a mutation in the protospacer region and the remaining nine CEMs had mutations in PAM sequences (four in the CRISPR1 PAM and five in the CRISPR3 PAM). Each of the five second-order CEMs that evaded the immunity of the second-order BIMs had acquired a second mutation, as expected. One had acquired a second mutation in a protospacer, two had mutations in the PAM, one contained one mutation in the protospacer and another in the PAM, and the last one had an insertion in the PAM. Taken together, these sequencing data are consistent with previous results indicating that CEMs are the product of mutations in the protospacers and/or PAMs which enable these viruses to circumvent CRISPR–*cas* acquired immunity [7], [8].

### Population dynamics

#### Optical density data and the criteria for resource- and phage-limited cultures

Although we estimated cell and phage densities for specific samples by serial dilution and plating, much of our inference about the population dynamics of these bacteria and phage comes from optical density (OD 600 nm) data. In these experiments, we follow the changes in OD of cultures of bacteria and phage with frequent sampling for 8 or so hours. To illustrate how the changes in optical density are related to changes in cell density and what we would anticipate for these changes in resource- and phage-limited cultures, we inoculated 4 ml broth with 40 µl of an overnight culture of WT cells (for a final concentration of approximately 2×10^6^ cells per ml), with different initial concentrations of phage, and without phage for the control.

As anticipated (intuitively as well as from the model), the time before the bacterial density declines due to the phage is inversely proportional to the initial density of these viruses (Figure S2). As predicted by the model, the optical densities of the cultures with phage converge to a level well below that of the phage-free control. However, the times required for these convergences to occur in our experiments are greater than predicted.

#### Phage and sensitive bacteria

**Figure 5:**
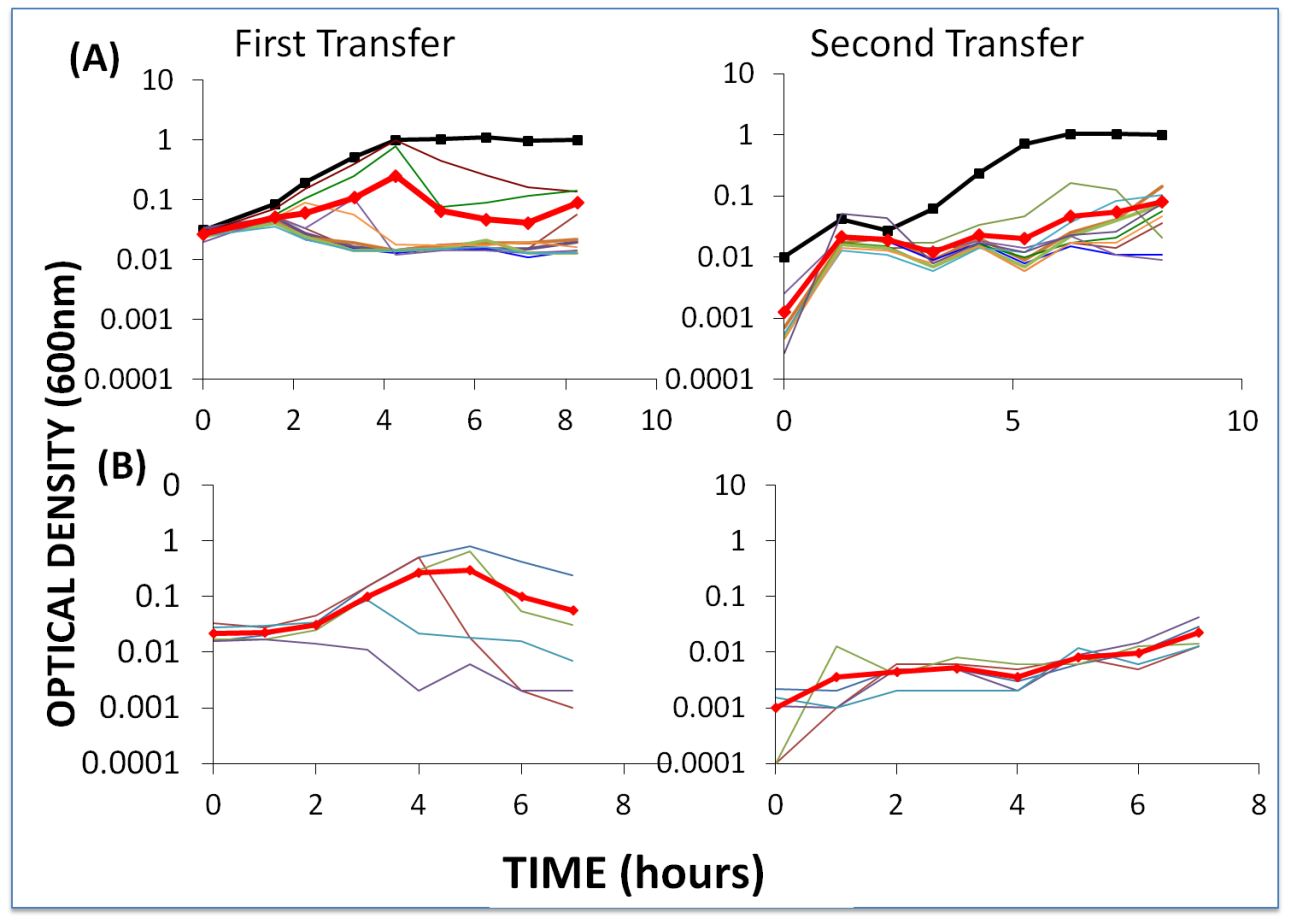
Changes in optical density of cultures with *S. thermophilus* and the corresponding phages to which these WT cells or BIMs are sensitive. The first transfer was initiated with 40 µl of an overnight culture (for approx. 2×10^6^ cells per ml) and phage (approx. 2×10^6^ per ml) in 4 ml LM17Ca medium. The second transfer was initiated by adding 40 µl of the 24-hour first-pass cultures to 4 ml fresh LM17Ca. The heavy black lines with square tick marks indicate phage-free WT controls. The heavy red lines with diamond tick marks are the arithmetic mean ODs for the cultures with bacteria and phage. Panel A: WT *S. thermophilus* and WT phage (light blue) and nine first-order BIMs and their corresponding CEMs. Panel B: five second-order BIMs and their corresponding CEMs.

To determine whether the failure of BIMs to emerge when sensitive bacteria are confronted with phage is a general result, we mixed 2×10^6^ WT cells, first-order BIMs, and second-order BIMs with approximately 2×10^6^ WT phage, first-order CEMs, and second-order CEMs, respectively, and followed the changes in OD for two transfers. The results of these experiments are presented in Figure 5.

During the first transfers, the OD of the cultures with phage initially rose and then declined. In the second transfer, these ODs remained at least an order of magnitude less than those of the phage-free controls. The initial densities of phage in the first transfer cultures ranged from 1.2×10^6^ pfu/ml (BIM4) to 3.4×10^7^ pfu/ml (BIM10). With the exception of BIM1, the estimated cell densities in these cultures with phage were between 6.0×10^4^ cfu/ml (BIM7) and 1×10^7^ cfu/ml (BIM6). In the absence of phage, the average 24-hour density of wild type cells and BIMs exceeded 2×10^8^ cfu/ml. This experiment was repeated at least three times and, qualitatively, the same results were obtained; the cultures with phage remained at optical densities in the range we consider to be phage- rather than resource- limited.

To determine whether BIMs resistant to the phage in these cultures emerged, a similar experiment was performed without frequent sampling. Single colonies were taken from each of the first- and second-order BIM-CEM cultures at end of the first transfer. These colonies were re-streaked and grown up in liquid culture and, as with the wild type bacteria, were spot tested for resistance to the corresponding CEMs. While we can't rule out minority populations of BIMs resistant to these phages, all of the colonies tested were sensitive to the phages.

#### Resource- or phage-limited densities

In the preceding, we use the phrase “phage-limited” to describe situations where the ODs of bacterial cultures with phage remain substantially less than those they would achieve were the phage not there and the bacteria limited by the availability of resources. Implicit in the phrase “phage-limited” is the assumption that there is an abundance of resources that could be used by the bacteria for growth were their densities not limited by the phage or products of phage replication. To test this phage-limited hypothesis, we inoculated batch cultures with wild type, first-order, or second-order BIMs and phage capable of growing on these bacteria. After 24 hours, we transferred the cultures to fresh medium (1/100 dilutions) and maintained the original and diluted cultures, estimating the optical density each day. All were in the “phage-limited” range (approximately 0.2 or lower). At 72 hours, we divided these 4 ml cultures in half. To one half we added 40 µl of an overnight culture of another industrial *S. thermophilus* strain (SMQ-301), which is unrelated to DGCC7710 and is insensitive to phage 2972. After 24 hours of incubation, we estimated the OD of the cultures with and without SMQ-301. At that time, the original culture was 72 hours old and the diluted culture 48 hours old.

The ODs of the cultures with SMQ-301 are substantially greater than those without these distinct cells. The extent of this residual growth is inversely proportional to the density of the culture to which these SMQ-301 cells are added. The linear regression coefficient for 31 pairs of cultures with and without SMQ-301 is −2.53, (F(1,29) = 24.6, p<2.7×10^−5^). We interpret these results as direct evidence that the phage, rather than resources limit the density of bacteria in cultures maintained at lower optical densities (see Table S2).

#### Population dynamics of phage and BIMs with single-spacer resistance

As suggested earlier, one reason BIMs do not ascend and take over cultures with sensitive bacteria and phage (Figure 2 and Figure 5) might be that they are not generated in the sensitive population (i.e. the rate of BIM formation is too low). If BIMs are originally present in cultures of bacteria sensitive to the dominant population of phage, it would seem that unless they are countered by phage mutants (CEMs) capable of replicating on them, these resistant BIMs would ascend to dominate the cultures. Moreover, because the phage would adsorb to these resistant BIMs, phage densities would be expected to wane and eventually the culture should be cleared of phage. If, however, CEM phages are initially present or are generated during the course of replication, these phages would be expected to ascend and the BIM population would be reduced and potentially eliminated. To illustrate these predicted results, we use our model and consider two situations: one in which a BIM is the only bacterial population and is resistant to all but a single phage particle, and one where there are no CEM phage and initially equal densities of the BIM and WT cells upon which the phage can grow and produce CEMs (Figure S3).

First scenario: in the absence of bacteria upon which it can replicate, the density of P_0_ phage continues to decline because of adsorption to the immune B_1_. The rare P_1_ population, which can grow on B_0_ and B_1_, ascends and kills off the B_1_ population (Figure S3A). Second scenario: although they are not initially present, during the course of replication on B_0_, the CEM phage P_1_ arise and ascend. Both populations of bacteria are eliminated and both wild type and CEM phage persist (Figure S3B).

**Figure 6:**
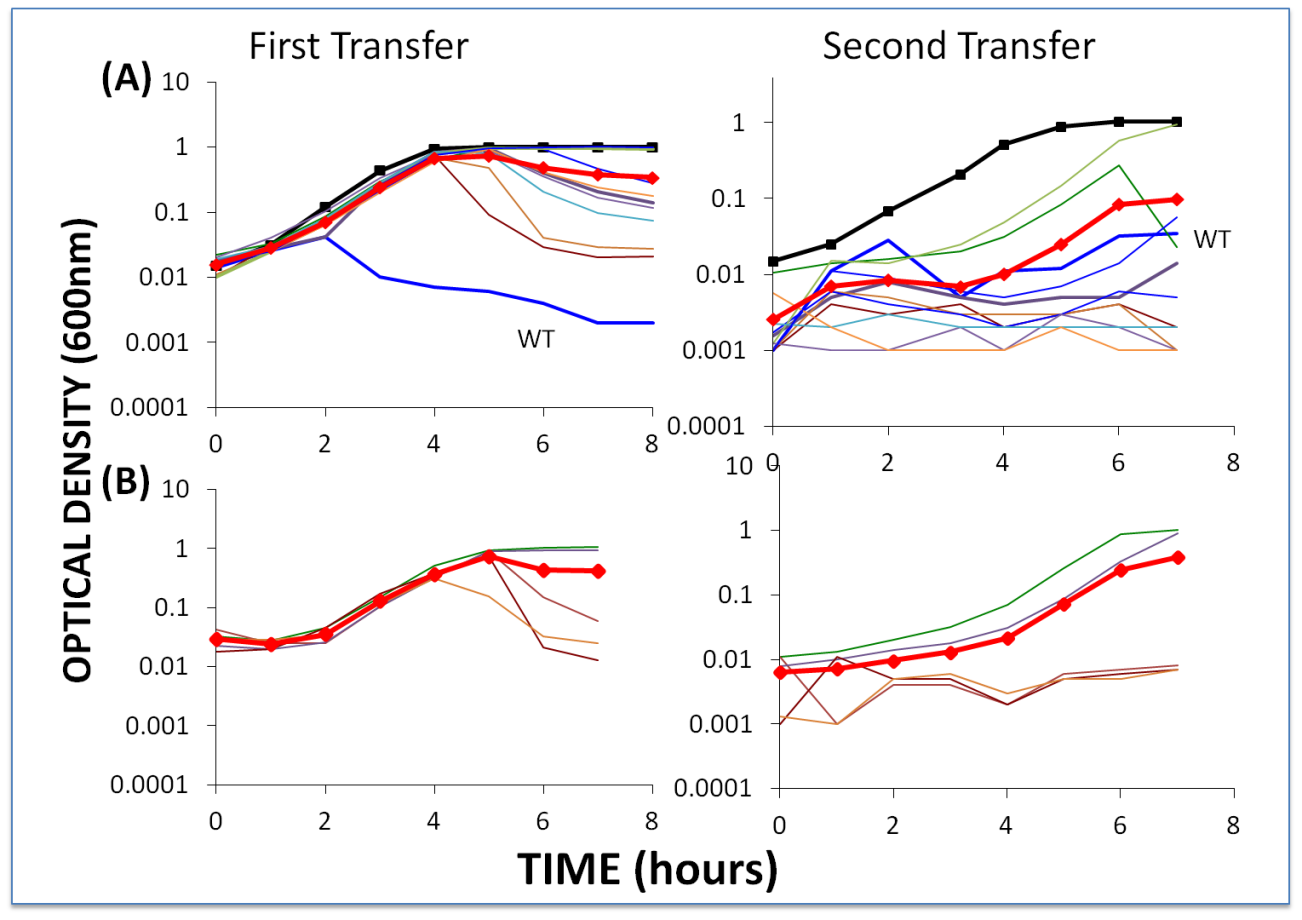
Changes in optical density of cultures with *S. thermophilus* and high initial densities of phage (approx. 5×10^6^/ml) to which the BIMs were resistant. The first transfers (left) were initiated with 40 µl of overnight bacteria and phage in 4 ml LM17Ca medium. The second transfers (right) were initiated by adding 40 µl of the 24-hour first-pass cultures to 4 ml fresh LM17Ca. Panel A: WT phage with WT cells (medium-weight blue line) and 10 first-order BIMs. Panel B: five second-order BIMs and their corresponding first-order CEM phages. The heavy black lines with square tick marks are phage-free WT controls and the heavy red lines with diamond tick marks are the arithmetic mean densities of all the cultures with phage.

To test these hypotheses for situations where the bacteria are resistant to the dominant population of phage, we inoculated cultures with first-order BIMs and WT phage and second-order BIMs with their corresponding first-order CEMs, e.g. CEM2 with BIM2^2^ (approx. 2×10^6^ phage and cells per ml). As in the experiment depicted in Figure 5, we followed the changes in optical density and at 24 hours diluted the cultures 100 fold in LM17Ca and followed the changes in OD for this second transfer. The results of this experiment are presented in Figure 6.

Two lines of evidence support the hypothesis that the failure of these BIMs to ascend to the OD level of phage-free cultures and the decline in their densities can, at least in part, be attributed to the emergence of CEM phage capable of replicating on these resistant cells. First, based on our estimates of the frequency of CEMs in WT lysates (Figure S1), we would anticipate one or more CEMs in the original inoculum. Second, and more directly, the phage recovered at 48 hours generated clear zones on lawns of WT cells and lawns of their respective BIMs. For example, clear zones were noted when lysates from the culture initiated with WT phage and BIM1 were spotted on BIM1 lawns and when cultures initiated with CEM2 and BIM2^2^ were spotted on BIM2^2^ lawns.

**Figure 7:**
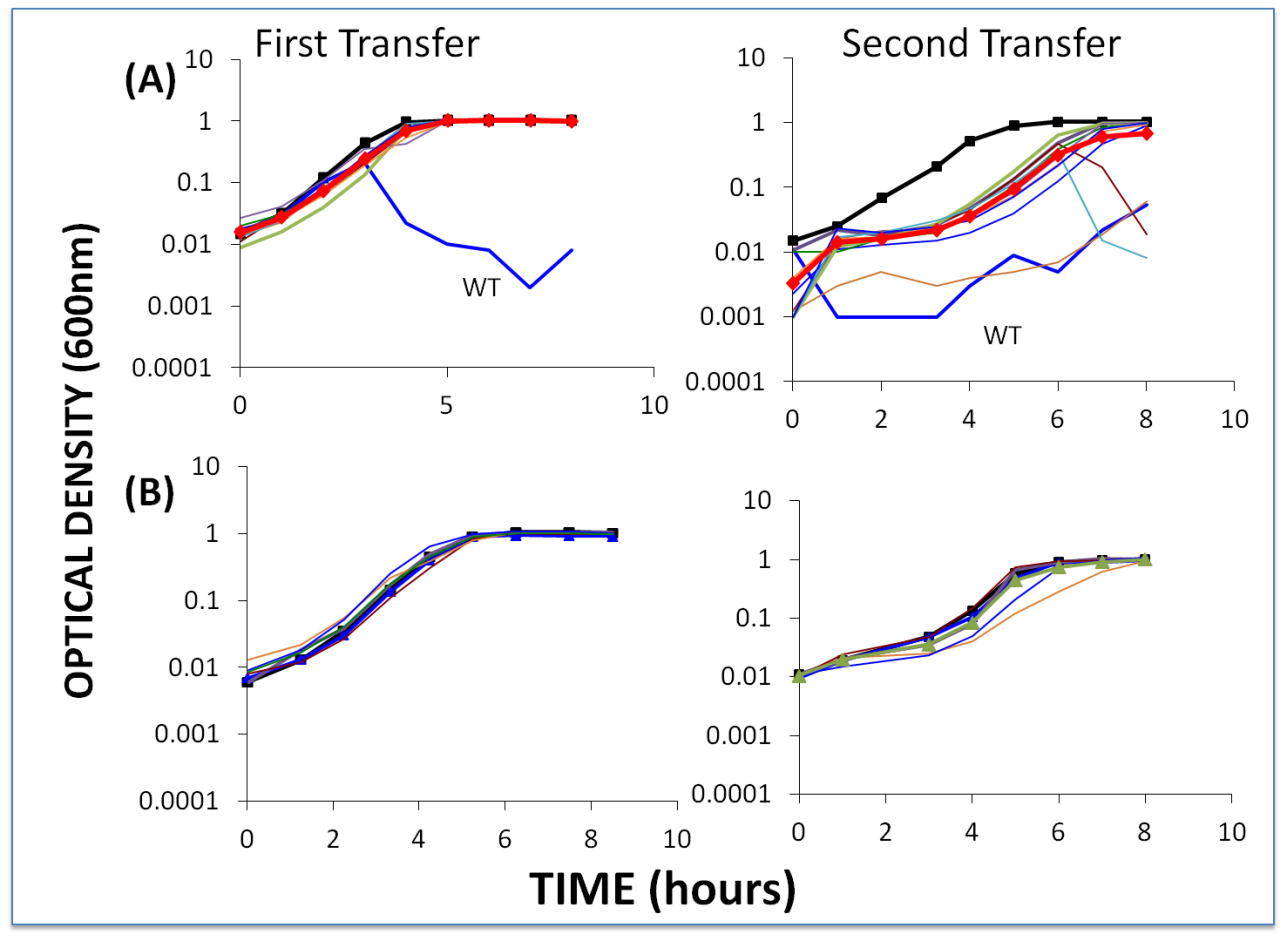
Changes in optical density of cultures with *S. thermophilus* and low initial densities of WT phage. The first transfer was initiated with 40 µl of an overnight culture of bacteria (approx. 2×10^6^ cells per ml) and WT phage (approx. 2×10^4^ ml) in 4 ml LM17Ca medium. The second transfer was initiated by adding 40 µl of the 24-hour first-pass cultures to 4 ml fresh LM17Ca. Panel A: WT phage with WT cells (medium-weight blue line) and 10 first-order BIMs. Panel B: WT phage with five second-order BIMs. The heavy black lines with square tick marks are phage-free WT controls. The heavy red lines with diamond tick marks are the arithmetic mean densities of all BIM cultures with phage.

To further test the hypothesis that CEMs will emerge and ascend only when single-spacer resistant BIMs are exposed to phage populations that are large enough to have CEMs bearing mutations in the protospacer (or PAM) targeted by the BIMs, we performed the above experiment with low initial densities of phage. Based on the estimated frequencies of CEMs, with the low initial numbers of phage in these cultures (approximately 2×10^−4^) we would not anticipate preexisting CEMs with the needed mutations. As a positive control, we performed these experiments with second-order BIMs and low densities of WT phage (Figure 7). Since second-order BIMs are protected by two spacers, the generation of second order CEMs – which would require mutations in two different protospacers in order to permit infection of second-order BIMs – is not anticipated. The results of this assay are presented in Figure 7B.

The second-order BIMs with low densities of WT phage (Figure 7A) behaved as anticipated. Moreover, in our enrichment experiments with 100 µl of overnight cultures of sensitive bacteria added to growing cultures of second-order BIMs, we were unable to find CEMs capable of growing on the second-order BIMs. And due to adsorption to the second-order BIMs (and intracellular cleavage of their genome by the CRISPR–Cas system), by the end of the second transfer there were few (less than 10^2^) WT phage in these cultures. Indeed, for three of the five second-order BIMs, we were unable to recover any phage at the end of the second transfer in enrichment experiments when 100 µl filtrates added to cultures with WT cells. In essence, phage cannot initiate an arms race when confronting BIMs that have acquired two or more spacers matching parts of the phage genome, despite the high rate of CEM generation.

The results obtained with low densities of WT phage and first-order BIMs are inconsistent with what our model predicted. Although there was no evidence for CEMs evolving in the majority of cultures with first-order BIMs and low densities of WT phage, they did emerge in two of the 10 first-order BIM cultures (BIM2 and BIM8). Furthermore, in a replicate of this experiment, CEMs emerged in 3 out of 10 cultures with first-order BIMs (BIM2, BIM3 and BIM7). In all cases where these CEMs emerged, the cultures became phage- rather than resource-limited. At this juncture we do not know why, in the second transfer, the growth of the bacteria in cultures for which we did not isolate CEM mutants (Figure 7A) was delayed relative to the phage-free controls. One possibility is that CEM mutants in these cultures delayed the replication of the bacteria, but were undetectable at 8 hours.

#### Population dynamics with first- and second-order BIMs with low densities of WT phage and WT cells

**Figure 8:**
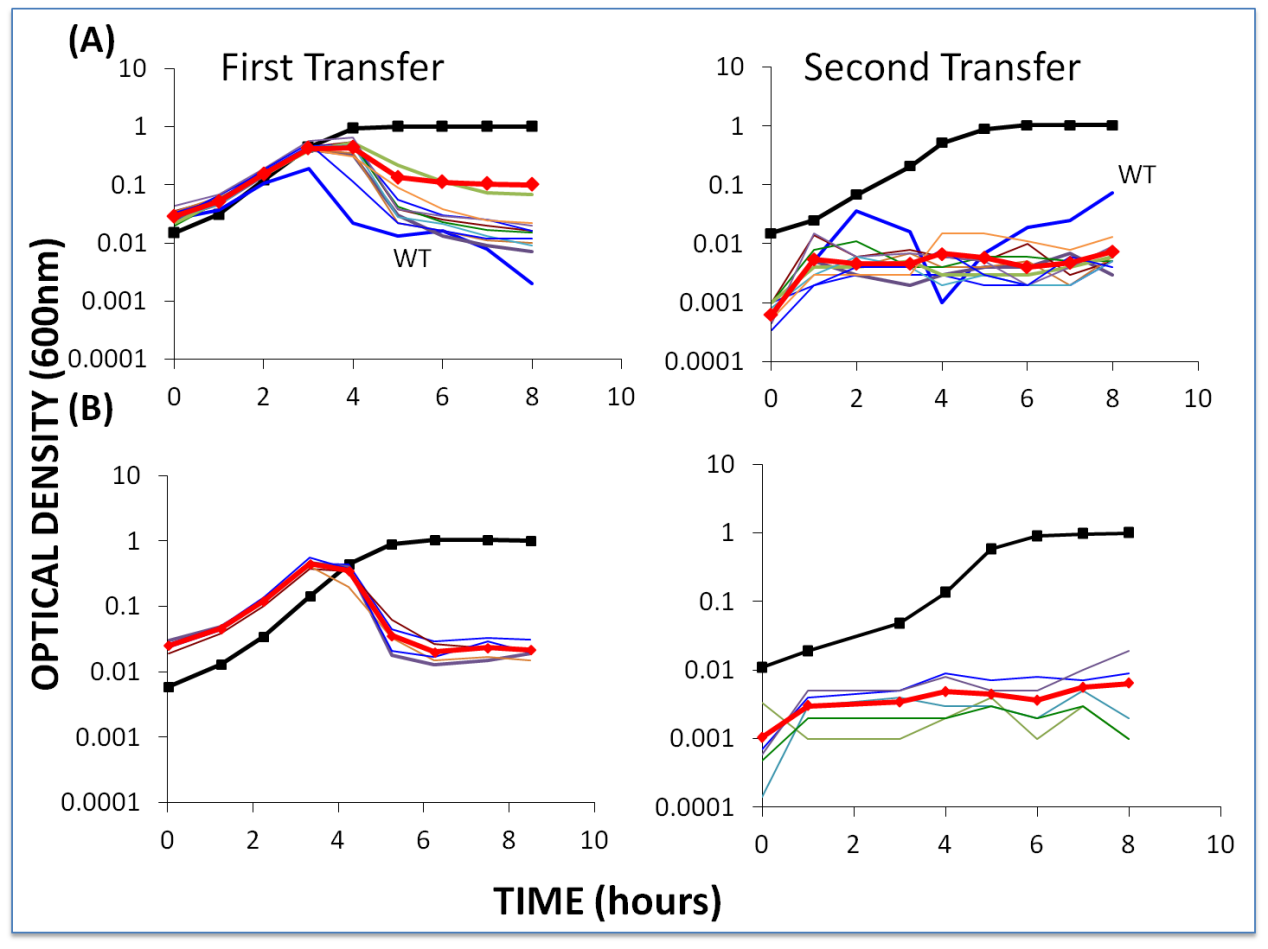
Changes in optical density of cultures with *S. thermophilus* first- and second-order BIMs and WT cells with low initial densities of WT phage. The first transfer was initiated with 40 µl of an overnight culture of each BIM (approx. 2×10^6^ per ml) and WT cells (approx. 2×10^6^ per ml) and low densities (approx. 2×10^4^ per ml) of WT phage in 4 ml LM17Ca medium. The second transfer was initiated by adding 40 µl of the 24-hour first-pass cultures to 4 ml fresh LM17Ca. Panel A: WT phage with WT cells and 10 first-order BIMs. Panel B: low densities of WT phage (approx. 2×10^4^ per ml) with WT cells and five second-order BIMs. The heavy black lines with square tick marks are the phage-free controls (WT cells in (A) and BIM2^2^ in (B)). The heavy red lines with diamond tick marks are the arithmetic mean densities of all the BIM cultures with phage.

It seems reasonable to assume that if bacteria with CRISPR–*cas* immunity were invading a habitat with phage present, there would also be sensitive bacteria upon which these phage would be replicating. To mimic this situation, we performed experiments with low initial densities of phage but also with WT cells at densities similar to those of the BIMs. The results of one of these experiments are presented in Figure 8.

Within the first transfer for both first- and second-order BIMs, the density of the bacteria declined to phage-limited levels. In accordance with our model, we would anticipate this result for the first-order BIMs due to the generation of CEMs in the course of replication of the phage on the wild-type host. Even if they were not present at the start of the experiment, first-order CEMs are likely to emerge whilst the phage are replicating on the WT cells. On the other hand, they would not be expected to emerge in the cultures with second-order BIMs. And, indeed, we have been unable to detect the presence of second-order CEMs at the end of the 2^nd^ transfer of the above experiment. As we discuss below, processes other than the emergence of second-order CEMs are responsible for the failure of second-order BIMs to ascend in these cultures with WT phage and WT cells. It should, however, be noted that, at least for the second-order BIMs, the phage-limited state observed for the first 8 hours of the second transfer was not maintained. At 26 hours, two of the five cultures in this second transfer were already as turbid as the phage-free controls and the remaining three were on their way to being so.

### Accounting for the deviations from the predictions of the model

At best, the mathematical model and computer simulations employed here are simplistic caricatures of the population dynamic and other processes occurring in these experiments. They are not intended or anticipated to provide quantitatively precise predictions of the changes in density of bacteria and phage observed in our experiments. Some quantitative deviation between experiments and theory is expected. Not expected, however, are major qualitative deviations. There were two of these. One is the failure of the phage to eliminate the sensitive bacteria. The other is the failure of BIMs to ascend and dominate cultures with an abundance of resources. We postulate these failures can be attributed to either the phage or the products of phage replication generating phenotypic immunity among sensitive cells (persistence) [37] and killing emerging BIMs.

**Figure 9:**
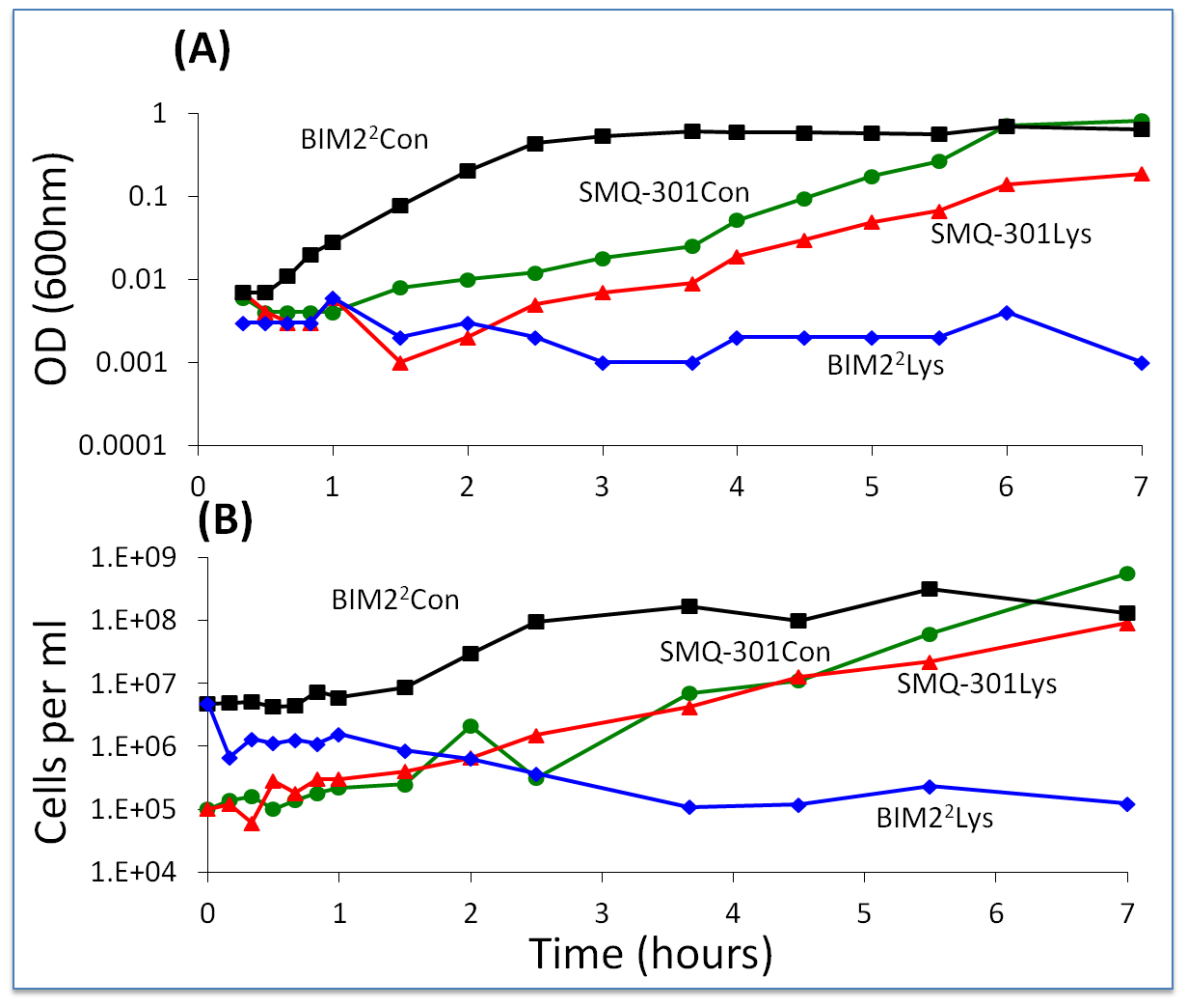
Changes in optical and viable cell density of *S. thermophilus* in cell-free lysates of wild-type phage. BIM2^2^ and SMQ-301 in a full concentration phage lysate (1.5×10^8^ pfu/ml) and an LM17Ca control (1/100 dilution). Con – LM17Ca control; Lys – cell-free phage lysate. Panel A: optical density. Panel B: estimated cell density.

One line of evidence in support of the hypothesis that something(s) in the phage lysate is preventing BIMs from ascending and also generating persisters comes from results obtained with 24-hour cell-free (filtered, rather than chloroformed) lysates of WT phage. *S. thermophilus* cells resistant to the phage (BIM2^2^ and SMQ-301) were added to these lysates and the optical and viable cell densities were followed with frequent sampling. In this experiment, 40 µl of BIM2^2^ or SMQ-301 from overnight cultures were added to 4 ml of the phage lysate. As phage-free controls, these bacteria were added to 4 ml LM17Ca (Figure 9). Cells of SMQ-301 were able to grow in these WT phage lysates, thereby indicating that there were plenty of resources. However, the viable cell density of the BIM2^2^ cells declined. At 24 and 31 hours, the optical density of the BIM2^2^ culture in the phage lysate was less than 0.02 and the viable cell densities were, respectively, 4.7×10^5^ cfu/ml and 1.2×10^6^ cfu/ml. At 48 and 54 hours, these viable cell densities were, respectively, 5.6×10^5^ cfu/ml and 2.1×10^5^ cfu/ml. The sensitivity of BIM2^2^ to the phage and/or molecules in the WT lysates was not shown by SMQ-301.

A second line of evidence in support of the hypothesis that phage or the products of their replication or lysis of infected cells prevent the ascent of BIMs is the results of our experiments with WT phage in cultures with first and second-order BIMs. Batch cultures inoculated with low densities of WT phage and bacteria that are resistant to those phages by one or two spacers become resource-limited. However, if we allow for considerable phage replication by adding WT cells, cultures with first- and second-order BIMs become phage-limited (Figure S4).

We have yet to characterize the agent(s) responsible for killing and/or inhibiting the growth of infected BIMs. However, preliminary results from experiments with lysates prepared from French Press extracts suggest that this agent is likely to be present within the cells and need not be a product of phage infection. The growth rates of resistant BIMs are markedly reduced when cultured in mixtures of medium and French Press extracts of phage-free cultures of wild type cells (Text S1). In this interpretation, this agent is released as a product of lysis of the cells by the phage.

### An extended model

*Never believe an experiment until it has been confirmed by a theory*. (attributed to Sir Arthur Eddington)

To explore, in a more quantitative way, whether the mechanisms postulated above, where products of phage replication generate phenotypically resistant persisters and kill BIMs, can account for the observed dynamics, we use an extension of the basic model described in the Theoretical Framework. In this extension, we consider three bacterial populations with densities *B_0_*, *B_1_*, and *B_2_*, and two populations of phage, *P_0_* and *P_1_*, respectively corresponding to WT, first- and second- order BIM bacteria, and WT and first-order CEM phage. For convenience, we assume there are agents of collective concentration *LY* µg/ml that kill resistant cells and provide transient immunity to sensitive bacteria (persistence effect [38], [39]). Since the effects of *LY* in killing resistant cells and generating persisters are governed by separate parameters, they can be at least two different compounds. These agents are produced at a rate proportional to the product of the densities of replicating phage and the corresponding sensitive bacteria:
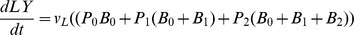
where v_L_ is a rate parameter for the production of *LY*. We assume that the rates of growth of the bacteria now depend on the concentration of *LY*, as well as the resources, *R*. For example, the per capita growth of B_0_ is now 
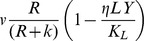
 where *ν* is the maximum growth rate of this strain, *k* the Monod constant, η the maximum rate of killing by *LY*, and *K_L_* the concentration of *LY* where killing is at its maximum rate. In the simulation *LY/K_L_* is set equal to 1 when *LY* exceeds *K_L_*. To account for phenotypically resistant B_0_ and B_1_ bacteria, we assume there are populations of non-dividing, persistent cells of densities BP_0_ and BP_1_ bacteria per ml that are produced from their respective dividing populations at a rate proportional to *LY/K_L_* and a constant (*g*) and return to the dividing state at a constant rate (*h*). For example, the rate of change in the density of the wild type (B_0_) population is now
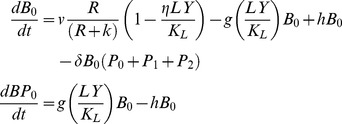
The Berkeley Madonna simulation used for this extended model can be obtained from www.eclf.net/programs.

**Figure 10:**
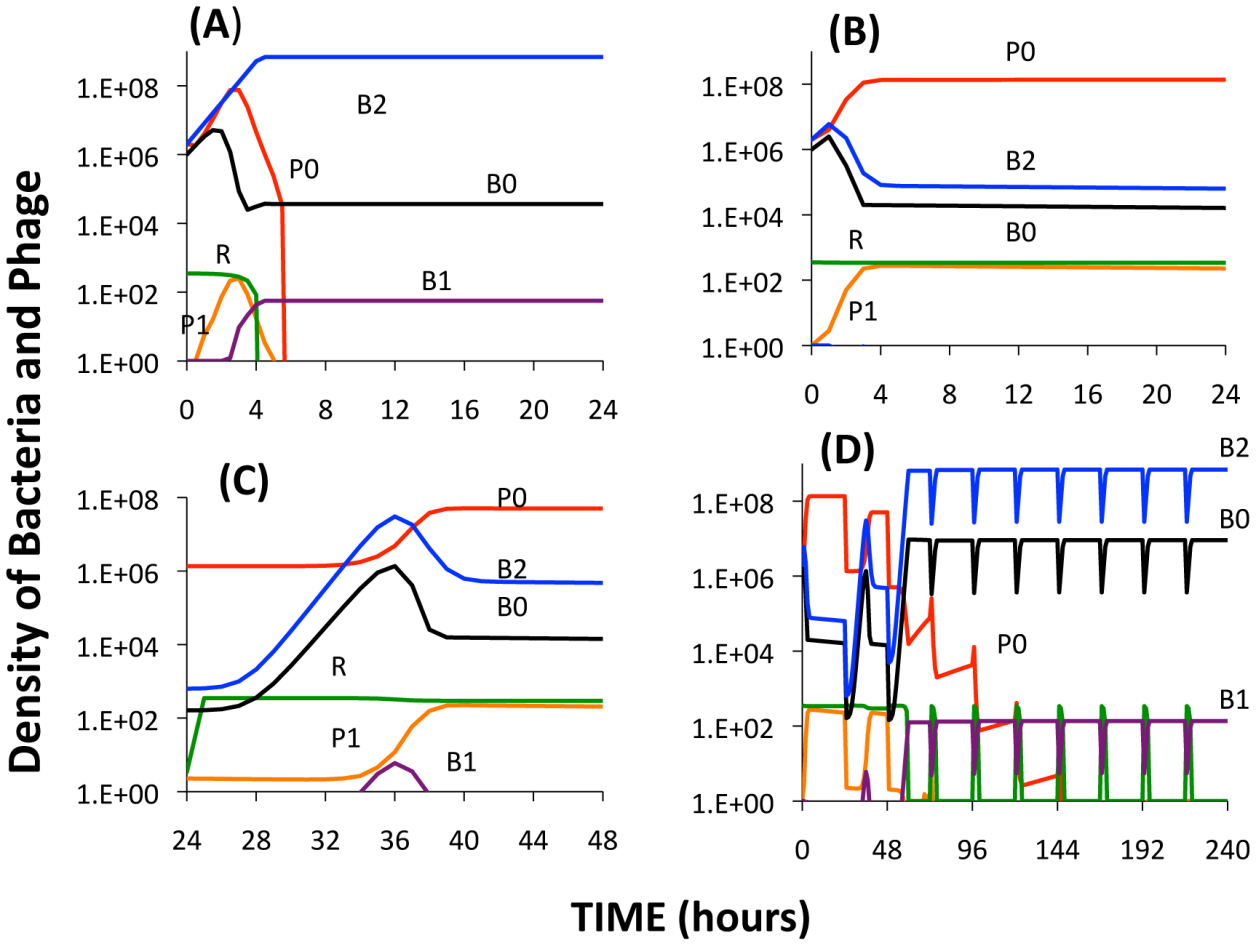
Simulation results of the extended model. Changes in the densities of bacteria and phage in cultures inoculated with WT phage (P0), second-order BIMs (B2), and WT cells upon which the phage can replicate (B0). The first-order BIMs (B1) and first-order phage (P1) are respectively produced by spacers added to the CRISPR region and generated by mutation, with probabilities of 10^−6^ per cell or phage per hour. The variable R is the concentration of the limiting resource. The bacterial growth and phage infection parameters in these simulations are the same as those in Figure 2. Save for (A) the parameters for the production and action of LY and persistence are as follows: v_L_ = 10^−6^, *K_L_ = 10^6^, η = 1.4, g = h = 0.01*. The initial concentration of the resource at the start of a transfer is 350 µg/ml. The cultures were initiated with 2×10^6^ B0 and B2 cells and 2×10^6^ P0 phage. Panel A: No LY produced (v_L_ = 0). Panel B: The first transfer of the culture. Panel C: The second transfer. Panel D: sequential transfers (approx. 2×10^4^).

In Figure 10, we present the results of simulation experiments initiated with wild type phage (P_0_), wild type bacteria (B_0_), and a second-order BIM (B_2_). The population growth, initial resource concentration and phage infection parameters used in this simulation are same as those used in earlier simulations and thus in the range estimated in these experiments. The parameters for the production of *LY* and its action are arbitrary and chosen to illustrate the principle rather than fit specific data in any but a qualitative way.

In the absence of the killing effect, Figure 10A, the B_2_ population ascends and the phage are eliminated. The B_0_ population continues to be maintained, B_1_ bacteria and P_1_ phage are produced, and within short order the population becomes resource-limited.

During the first transfer of the simulations with the killing effect (Figure 10B), the P_0_ phage ascend and the densities of the B_0_ and B_2_ populations decline and the community becomes phage limited. While the second transfer (Figure 10C) also ends in a phage - limited state, the relative density of B_2_ is now greater. In subsequent transfers (Figure 10D), B_2_ ascends to dominance, phage are eliminated, and the community becomes resource-limited. As a consequence of the serial passages, the molecule killing BIMs is diluted out and the BIMs ascend. For the arms race to continue, the phage would have to generate CEMs capable of replicating on the dominant population of BIMs.

### Preliminary supporting experimental results

*While data may be a crutch to the insecure, really self-confident scientists subject their hypotheses to tests that will reject them*. (www.eclf.net)

**Figure 11:**
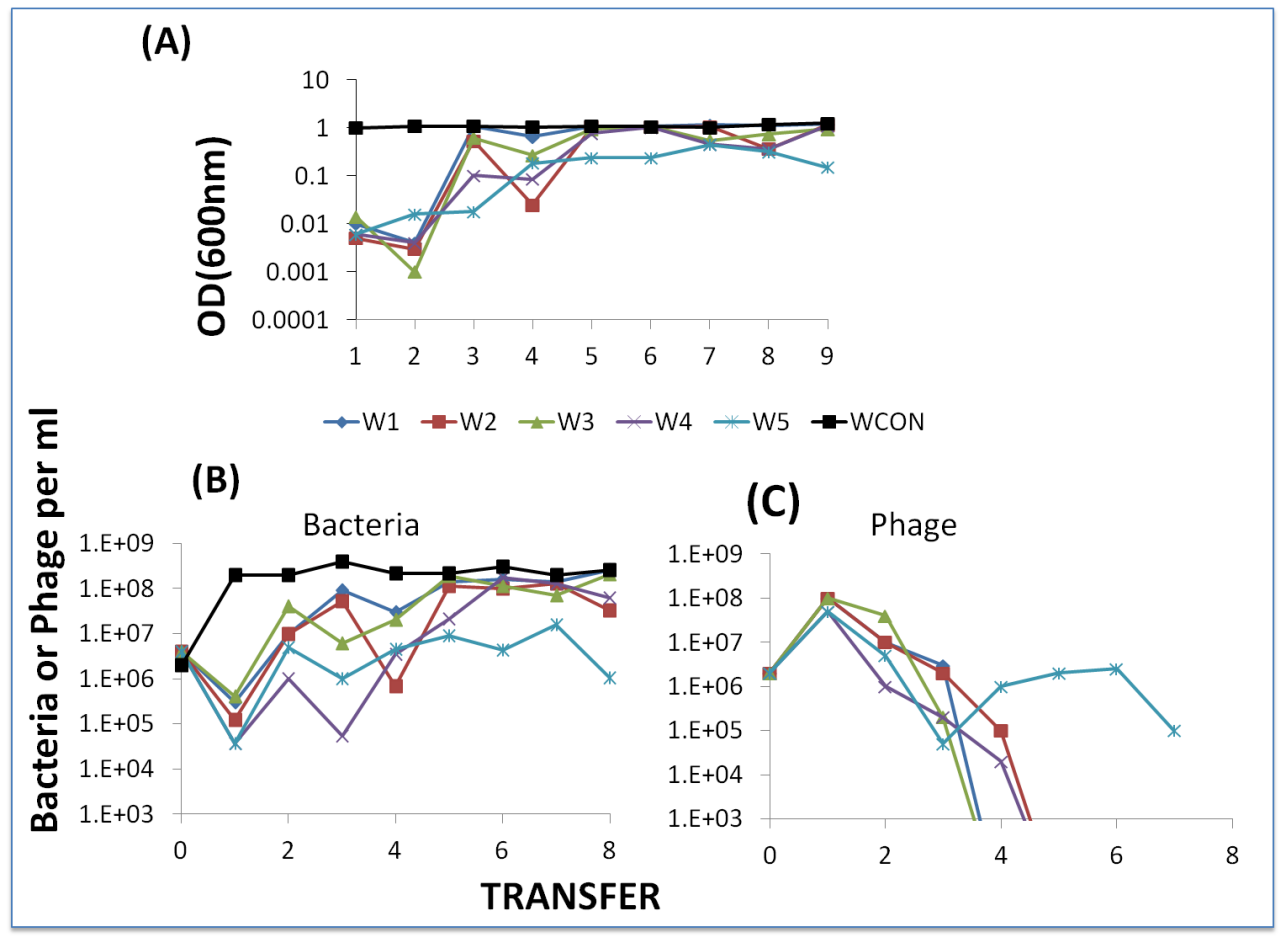
Serial transfer experiment initiated with WT phage and WT cells. Panel A: Optical densities at the end of successive passages. Panel B: estimated viable cell densities at the end of successive transfer. Panel C: estimated free phage densities at the end of successive transfers.

If the above postulated effect of dilution in serial passage is valid, in successive transfers of experiments initiated with WT cells and WT phage, BIMs should emerge. And, unless they are countered by CEM phage, they will ascend to dominance and the phage will be lost. To test this hypothesis, we mixed WT cells with phage and each day transferred the culture to fresh medium (40 µl into 4 ml). We initiated six separate cultures from the same mixture of approximately 2×10^6^ cells and phage. As a control we serially transferred a phage-free WT culture. At the end of each transfer, we estimated optical densities of these cultures and, by serial dilution and plating, the viable densities of bacteria and free phage. The results of this experiment are presented in Figure 11.

We interpret the results of this experiment to be consistent with the hypothesis that as a consequence of successive dilutions of the compound killing BIMs, the resistant mutants ascend. Moreover, in 5 of the 6 cultures in which BIMs emerged, phage were lost and could not be recovered even with enrichment with WT cells (i.e. the arms race was terminated). Additional serial transfer experiments should be performed with different starting conditions and possible different media (perhaps milk) to explore the generality of this observation about the limit to a BIM-CEM arms race.

## Discussion

We interpret the results of these experiments with *S. thermophilus* DGCC7710 and its virulent phage 2972, and the associated computer simulations, as support for the hypotheses that the CRISPR–*cas* systems can (i) prevent the invasion of phage into established communities of bacteria; (ii) enable a population of bacteria to become established and be maintained in communities with phage; and (iii) promote an arms race between phage-mediated selection for CRISPR–*cas* acquired immunity (BIMs) and immunity-mediated selection for CEMs capable of replicating on these BIMs. However, these experimental and computer simulation results indicate that the conditions under which these ecological and (co)evolutionary outcomes occur are restrictive in ways that are not predicted by the canonical view of phage infection and current understanding of CRISPR–*cas* immunity.

Although not surprising to the molecular biologist (RB) and microbiologist (SM) among us, it was particularly gratifying for the population-evolutionary biologists on this team (BRL and MB) to realize that the perspective of CRISPR–*cas* adaptive immunity one gleans from the simplified diagrams of reviews is a reality for the *S. thermophilus* DGCC7710 – phage 2972 system. Bacterial mutants resistant to WT phage (BIMs) were readily isolated. The resistance of all 10 BIMs examined can be accounted for by the acquisition of unique 30 base pair sequences (spacers) that correspond to regions of the phage genome. Phage capable of replicating on these first-order BIMs (CRISPR Escape Mutants, or CEMs) were also readily isolated. As anticipated from the canonical view of CRISPR–mediated immunity, the host range of these resistant viruses was restricted to the BIMs on which they evolved and their wild type ancestors, and can be attributed to mutations in protospacers (or PAMs) corresponding to the DNA sequences in the spacers acquired by their respective first-order BIMs. This iterative process of CRISPR–*cas* acquired immunity in the bacteria and CRISPR–escape mutation in the phage was also observed for the five second-order BIMs isolated from these first-order BIMs and their respective second-order CEM phage. How many orders of BIMs and CEMs can be generated by this iterative process of spacer acquisition by the bacteria and protospacer mutation in the phage remains unclear at this time and is a subject for future study.

Other than to say that first and second-order BIMs “are readily isolated,” for the reasons discussed below, we cannot provide estimates of the actual rate at which this phage resistance is acquired. What is clear is that CEMs are found at frequencies of 10^−6^ or greater in lysates. This seemingly high rate of CRISPR–escape mutation is particularly surprising because only a small fraction of the total sequence space in the protospacers-PAM region is responsible for generating these CEMs. It is tempting to speculate that as part of their adaptation to CRISPR–cas these phage have evolved a mechanism to elevate mutation rates.

On the other side, the results of our phage-host interaction experiments as well as our computer simulations indicate that there is more to the population and (co)evolutionary dynamics of phage and bacteria with CRISPR–mediated acquired immunity than can be inferred from the current perspective on this adaptive immune system. Moreover, qualitatively as well as quantitatively, there is more to these dynamics than can be inferred from models of the population dynamics of the interactions of bacteria and lytic phage based on the traditional view of phage infection [24], [25], [40].

First, although *S. thermophilus* BIMs can readily be picked up experimentally, even when present, these resistant bacteria do not reach measurable densities in liquid cultures with phage. For BIMs resistant through single spacers, one reason for this failure to ascend is the high rate of mutation to CEMs capable of replicating on and killing them. Most interestingly, CEMs emerge when the number of phage is too low to anticipate their presence in the original lysate or their generation during replication on sensitive cells. We postulate that the reason for this is that resistance due to a single spacer is not absolute and a fraction of the phage adsorbing to resistant cells replicate and produce CEMs. This interpretation is consistent with the previous observation that not all phage DNA molecules are cleaved by CRISPR–*cas* when infecting BIMs [8].

Another result not predicted by our simple model is that substantial densities of sensitive bacteria (in excess of 10^4^ cells per ml) survive their encounter with phage. We postulate that the survival of these genetically phage-sensitive cells can be attributed to phenotypic resistance [37] induced by a product of phage infection. A phenomenon of this type has been demonstrated in *Shigella dysentariae* infected with the phage T7 [38], [39]. In the interpretation of this process presented in the review by Barksdale [39], a phage enzyme is released as a product of phage infection and strips the phage receptors off of the surviving bacteria, thereby making them phenotypically immune to phage infection. Whether a similar mechanism is responsible for the phenotypic immunity to phage 2972 of *S. thermophilus* remains to be seen, and will constitute a worthy study in its own right.

The results of our experiments indicate that BIMs resistant to phage due to the acquisition of two spacers can establish communities where phage are present and prevent the invasion of phage mutants into established populations of bacteria. The conditions for these outcomes are, however, restrictive. If the community includes sensitive bacteria upon which the phage are replicating, neither first- nor second-order BIMs will become established. We postulate that this can be attributed to a product of phage infection that kills and/or inhibits the replication of BIMs. As a consequence of phage replicating on WT bacteria, there would be high densities of phage and correspondingly high concentrations of this product. While we have yet to characterize this product, there is evidence for phage 2972 bearing genes that code for an endolysin [34]. On the other hand, the results of additional experiment we performed indicate that the agent(s) responsible for inhibiting the growth of BIMs are not phage-encoded products, but rather released by the lysed bacteria (see the Text S1).

Models of the population dynamics of bacteria and phage that incorporate the persistence of sensitive cells and the killing of BIMs due to chemicals generated during the course of phage infection can account for the qualitative inconsistencies between the results predicted by the simple model and those observed experimentally. This extended model predicts that in the course of successive transfers, the concentration of this chemical would be reduced and BIMs resistant to the phage would ascend to dominate the culture. This prediction is supported by the results of our longer-term serial transfer experiments initiated with WT bacteria. Also consistent with this interpretation are the results of a recent metagenomic analysis of experimental populations of *S. thermophilus* and the phage 2972 [41].

### Hypotheses, caveats, and limitations

The results of this jointly theoretical and experimental study make at least five predictions about the contribution of CRISPR–*cas* immunity to the population and community ecology of bacteria and phage and the (co)evolution of these organisms.

1. A CRISPR–*cas* immune system can protect established populations of bacteria from invasion by phage, but only if all of the bacteria carry at least two spacers for resistance to the invading viruses.
2. Although CRISPR–mediated immunity can enable bacteria to invade established communities of bacteria and phage, the conditions for this outcome are restrictive: (i) the invading population of bacteria has to be resistant to the established population of phage by at least two spacers and (ii) the concentrations of inhibitory compound in the established community have to be low enough for the invading population of bacteria to replicate.
3. As a consequence of phenotypic resistance (persistence), communities of bacteria and phage will contain populations of bacteria that are sensitive to the phage.
4. Although BIMs and CEMs are readily generated at relatively high rates, a CRISPR–mediated BIM-CEM (co)evolutionary arms race may not ensue; BIMs to which CEMs are not or cannot be generated may ascend and dominate the bacterial population.
5. The acquisition of multiple spacers could be leveraged to effectively develop phage-resistant strains for industrial, biotechnological or food processes to protect against specific phage contamination.

Taken at large, this study predicts that the role of CRISPR–*cas* systems in the day-to-day ecology and evolution of natural communities of prokaryotes and their viruses is at best modest and more restrictive than anticipated from existing theory.

As compelling as we may consider these predictions to be, at this juncture we only see them as hypotheses that have not been fully tested. We have restricted this study to the dynamics of the early phase of the interactions between single populations of phage and bacteria with very active CRISPR–*cas* immune systems. While our models and experiments are appropriate for this phase of the association between these organisms, they are not sufficient for making predictions about the longer-term population and (co)evolutionary dynamics of these interactions. Most importantly, our models and experiments do not formally consider the potential diversity of BIMs and CEMs that can be generated at each level and the interactions between them (see, for example, [25]). How this diversity will play into the longer-term population and (co)evolutionary dynamics of these interactions and whether the bacterial populations will be limited by phage rather than resources remain to be seen. Particularly important in this regard is whether bacteria with other mechanisms of resistance to the phage will emerge and whether the phage will be able to generate mutants capable of replicating on these resistant cells (see [42] but also [43]). It may well be that the failure to observe bacteria with other resistance mechanisms in these experiments is due to the relatively high rate at which CRISPR–mediated immunity is generated in *S. thermophilus*.

Finally, in support of the prediction that CRISPR–mediated immunity plays a limited role in the day-to-day (rather than historical) ecology and evolution of prokaryotes and their viruses, is our choice of experimental system. *S. thermophilus* and the phage 2972 may well be an exceptional system for *in vitro* study of the population dynamics and evolution of phage and bacteria with CRISPR–mediated immunity. Not only is it arguably (surely, to those who work with it) the best-characterized naturally occurring CRISPR–phage system; with respect to the rate of acquisition of spacers and possibly the rate of generation of CEMs, it is the most active. It is an ideal system in which to observe CRISPR playing a prominent role in the ecology and evolution of prokaryotes and their viruses. To our knowledge, there is no evidence for naturally occurring CRISPR–*cas* systems of other organisms being as active in generating BIMs and CEMs as those of *S. thermophilus* and its phages. For these reasons, we conjecture that the CRISPR–*cas* systems of other bacteria, archaea and their phages would play an even less active role in the day-to-day ecology and evolution than anticipated from this study.

## Materials and Methods

### Bacteria and phage

For our experimental model, we selected the strain *S. thermophilus* DGCC7710 because it contains arguably the best-characterized CRISPR–*cas* functional system. *S. thermophilus* DGCC7710 [1], which bears four different CRISPR–*cas* systems, two of which are active (CRISPR1 and CRISPR3), was used as a host for the virulent phage 2972 [34]. We also used *S. thermophilus* SMQ-301 [44], which is resistant to 2972 as a non-host strain.

### Media and sampling methods

A single medium designated LM17Ca was used for all of the experiments. M17 (Oxoid) was supplemented with 0.5% lactose (L) and 0.05 M Calcium Borogluconate (Ca). For the soft/top agar and hard/bottom agar, LM17Ca was supplemented with 0.6% and 1.5% agar, respectively. Cell densities were estimated by dilution in 0.85% saline and plating on LM17Ca. To estimate phage titers, serially diluted samples were added to 1 ml of a late-log culture of *S. thermophilus*, put into 3 ml soft agar and poured onto LM17Ca plates.

### Isolation of BIMs and CEM phages

About 5×10^8^ *S. thermophilus* DGCC7710 cells were mixed with about 2.5×10^7^ of wild type (WT) phage 2972 and held for about 10 minutes. These mixtures were then added to 3 ml of LM17Ca soft agar, which was then poured over LM17Ca plates. Ten experiments identical to the above were run in parallel. All the plates were incubated at 42°C for 30 hours. Single colonies were removed from each plate and streaked twice to purify the BIM clones and remove contaminating phages. Resistance to WT phage 2972 was confirmed by spotting 10 µl (at least 5×10^5^ pfu) of the diluted lysate on soft agar lawns of the BIMs. Clear zones were scored as sensitive while turbid zones or few separate plaques were scored as resistant. From these experiments, we selected 10 BIMs (one per experiment), designated BIM1, BIM2, … BIM9, and BIM10. A similar procedure was used to randomly isolate five second-order BIMs from five of these first-order BIMs by spotting CEMs capable of replicating on these first-order bacteria. These were designated BIM2^2^, BIM3^2^, BIM5^2^, BIM7^2^, and BIM8^2^. CEM phages were isolated by mixing 100 µl of phage 2972 lysate with 1 ml of overnight cultures of first- or second-order BIMs. After 10 minutes these mixtures were added into 3 ml soft agar, poured onto LM17Ca plates, and incubated overnight at 42°C. Single phage plaques were picked from the plates the following day and added to 3 or 10 ml of LM17Ca. A few drops of chloroform were added to each tube to kill the remaining bacteria. These lysates were then vortexed and centrifuged. These single plaque lysates were then filtered (0.45 µm) and stored at 4°C. This single plaque isolation process was repeated two times to obtained purified phages. The CEM phages for the first-order BIMs (BIM1 through BIM10) were designated CEM1 through CEM10. The CEM phages for the second-order BIMs were designated CEM2^2^, CEM3^2^, CEM5^2^, CEM7^2^, and CEM8^2^.

### Assays for the presence of WT phage and CEMs

Two procedures were used to assay liquid cultures for the presence of WT phage and specific CEMs. One was a spot test: 10 µl of filtered or chloroform-treated cultures were either spotted directly or after serial dilution onto soft agar lawns of WT cells or first- or second-order BIMs. Either clear zones or turbid zones with specific plaques were considered to indicate the presence of phage capable of replicating on that bacterial cell line. In cases where negative results were obtained with this spot test, we used an enrichment procedure where 100 µl of the lysate was added to 4 ml of medium with 100 µl of overnight culture bacteria. The optical densities of these enrichment cultures were followed at 42°C and those that cleared were considered positive for phage capable of replicating on those host bacteria.

### Assays for the presence of BIMs

Two procedures were also used to test for the presence of bacteria resistant to wild type or CEM phages. One was a spot test: single colonies were streaked onto fresh medium to purify them from the phage in the cultures from whence they came. Single colonies were grown overnight at 42°C in liquid medium (1 ml) and then put into soft agar to be used as a lawn for spot testing with phage. For this we used the procedure and criteria for sensitivity/resistance described in the preceding paragraph. In some cases, the presence of resistant cells in samples was tested directly by adding 100 µl of phage and 100 µl of the cell culture to soft agar and looking for the presence of colonies.

### Molecular characterization of the BIMs and CEMs

The active *S. thermophilus* CRISPR loci, namely CRISPR1 and CRISPR3, were subjected to PCR, in order to test whether novel spacers had been acquired in BIMs following exposure to phage. Primers targeting the leader sequence and a unique WT spacer at the trailer end of CRISPR1, as well as a primer pair targeting the leader and the trailer end of CRISPR3, were used for PCR amplification. The names and sequences of these primers are, respectively: yc70 5′-TGCTGAGACAACCTAGTCTCTC-3′ and 89R6 5′-TCAGCAGATTGTCAAATCGG-3′ for CRISPR1 and F1 5′CTGAGATTAATAGTGCGATTACG-3′ and R2 5′-GCTGGATATTCGTATAACATGTC-3′ for CRISPR3. PCR amplifications were carried out as previously described [36], with Ambion Mastermix and the following PCR cycling: denaturation for 7 minutes at 95 C; followed by 35 cycles of 30 sec denaturation at 95C ; 30 sec hybridization at 53 C and 1 min extension at 72 C; followed by a final round of 5 min extension at 72 C. PCR amplicons were subjected to agarose gel electrophoresis for size analysis, purified using a Qiagen Kit, and subsequently subjected to Sanger sequencing using the same primer sets used for amplification. The sequencing results were visualized in EXCEL as previously described [36] in order to assess CRISPR repeat occurrence and visualize spacer content. In order to assess the protospacer sequences in the CEMs, targeted PCR amplifications followed by Sanger sequencing of the PCR amplicons were carried out for first and second generation CEMs as described previously [7], [8].

## Supporting Information

Figure S1 Relative frequency of CEM phage mutants: ratio of estimated number of phage from plaques on lawns of BIMs to that on wild type lawns. Panel A, CEMs for first-order BIMs (three independent experiments). Panel B, CEMs for second-order BIMs.
(TIF)

Figure S2 Observed and predicted changes in optical density of cultures with wild type *S. thermophilus* DGCC7710 and different initial densities of phage 2972. Panel A, predicted changes in optical density from model with the parameter values used in Figure 2. Panel B, observed changes in optical density anticipated from the model. To convert the population densities considered in this model into optical densities, we serially diluted a culture of a wild type *S. thermophilus* of known CFU density in LM17Ca and estimated the OD (600 nm) for the different dilutions. Using a polynomial fit to these cell density×OD data, we calculated the OD (600 nm) predicted for bacterial cell densities generated by the simulation. It should be noted that the leveling off of the OD around 0.005 is an artifact of the fitting procedure and the inability of OD data to estimate bacterial densities less than approx. 5×10^5^.
(TIF)

Figure S3 Simulation results showing changes in the density of bacteria and phage as well as the concentration of the limiting resource *r*. Panel A, initially a population of bacteria (B_1_) immune to the dominant population of phage (P_0_) and a single CEM mutant (P_1_) capable of replicating on B_1_. Panel B, initially equal densities of bacteria sensitive and immune to the P_0_ phage (B_0_ and B_1_) and no CEM phage (P_1_ = 0), but mutation to CEM at a rate μ = 10^−6^ per particle per burst. The population growth and phage infection parameters used for these simulations are those in the legend of Figure 2.
(TIF)

Figure S4 Optical density of cultures after 22 hours of incubation. Panel A, first-order BIMs (BIM1 and BIM2) with low densities (2×10^4^ pfu per ml) of WT phage or second-order BIMs (BIM3^2^ and BIM7^2^) with first-order CEMs (CEM3 and CEM7). Panel B, second-order BIMs with low densities (2×10^4^ pfu per ml) of WT phage with and without WT cells.
(TIF)

Table S1 List of new spacers acquired in CRISPR1 or CRISPR3 of *S. thermophilus* DGCC7710 and the corresponding protospacer regions in phage 2972.
(PDF)

Table S2 Mean and standard deviation in optical density (600 nm) with and without *S. thermophilus* SMQ-301.
(DOCX)

Text S1 Evidence for Phage-independent inhibition of replication of BIMs.
(DOC)
