# Supplementary figures and images for: The Population and Evolutionary Dynamics of Phage and Bacteria with CRISPR–Mediated Immunity
Source: PLoS Genet. 2013 Mar 14;9(3):e1003312. doi: 10.1371/journal.pgen.1003312 (PMC3597502; doi:10.1371/journal.pgen.1003312)

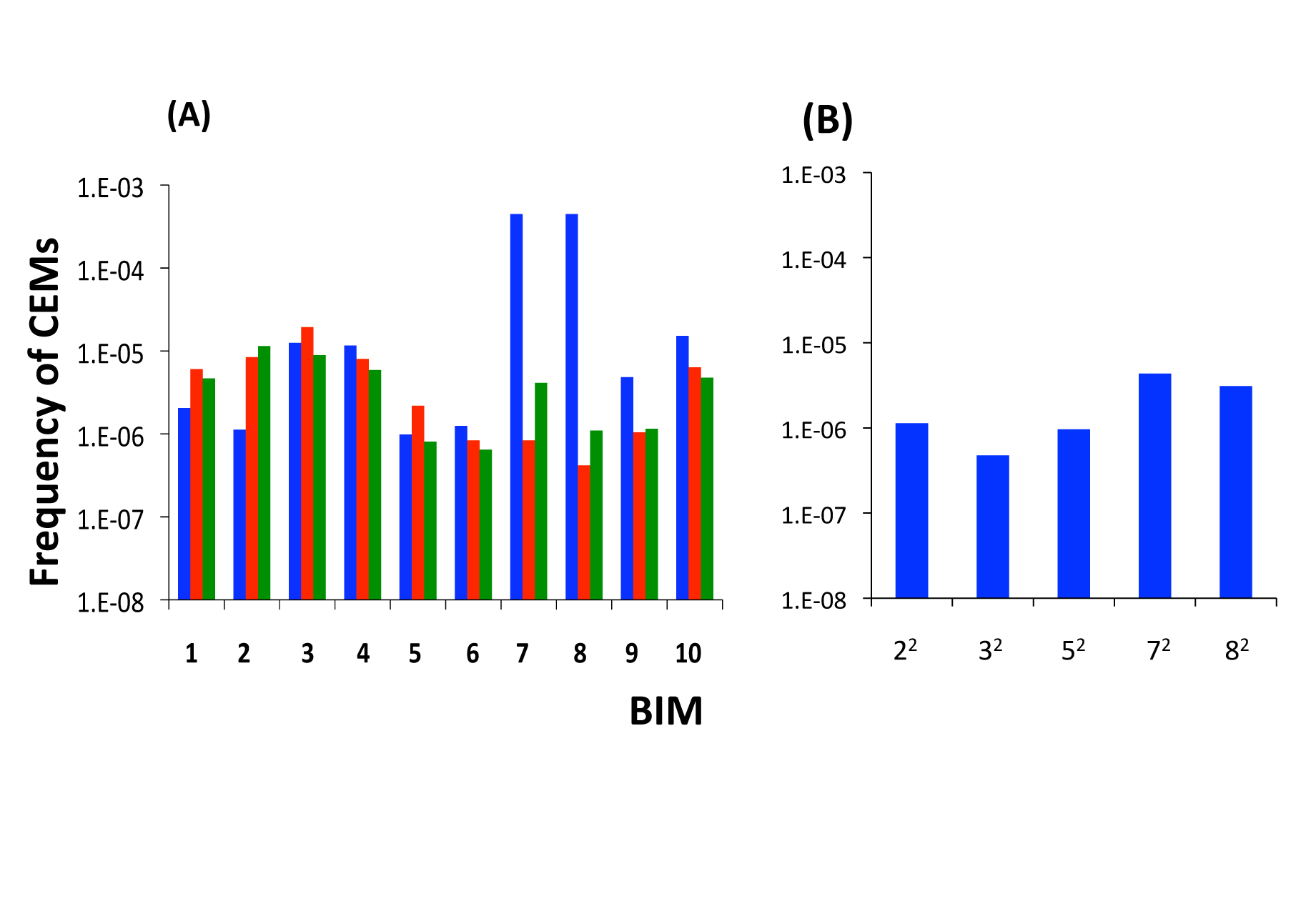

Supplement: Figure S1 — Relative frequency of CEM phage mutants: ratio of estimated number of phage from plaques on lawns of BIMs to that on wild type lawns. Panel A, CEMs for first-order BIMs (three independent experiments). Panel B, CEMs for second-order BIMs. (TIF) [file pgen.1003312.s001.tif]

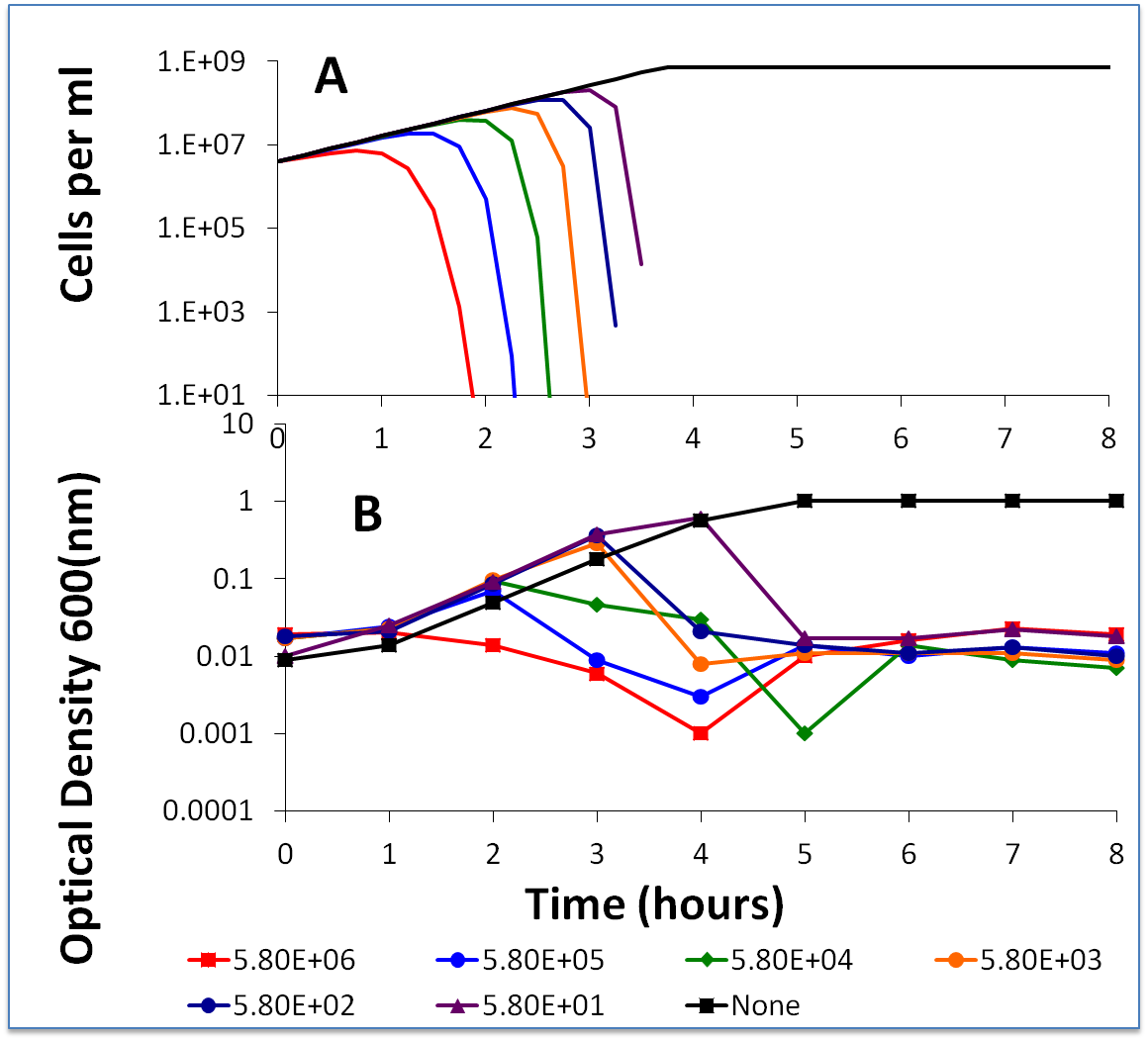

Supplement: Figure S2 — Observed and predicted changes in optical density of cultures with wild type S. thermophilus DGCC7710 and different initial densities of phage 2972. Panel A, predicted changes in optical density from model with the parameter values used in Figure 2. Panel B, observed changes in optical density anticipated from the model. To convert the population densities considered in this model into optical densities, we serially diluted a culture of a wild type S. thermophilus of known CFU density in LM17Ca and estimated the OD (600 nm) for the different dilutions. Using a polynomial fit to these cell density×OD data, we calculated the OD (600 nm) predicted for bacterial cell densities generated by the simulation. It should be noted that the leveling off of the OD around 0.005 is an artifact of the fitting procedure and the inability of OD data to estimate bacterial densities less than approx. 5×10^5^. (TIF) [file pgen.1003312.s002.tif]

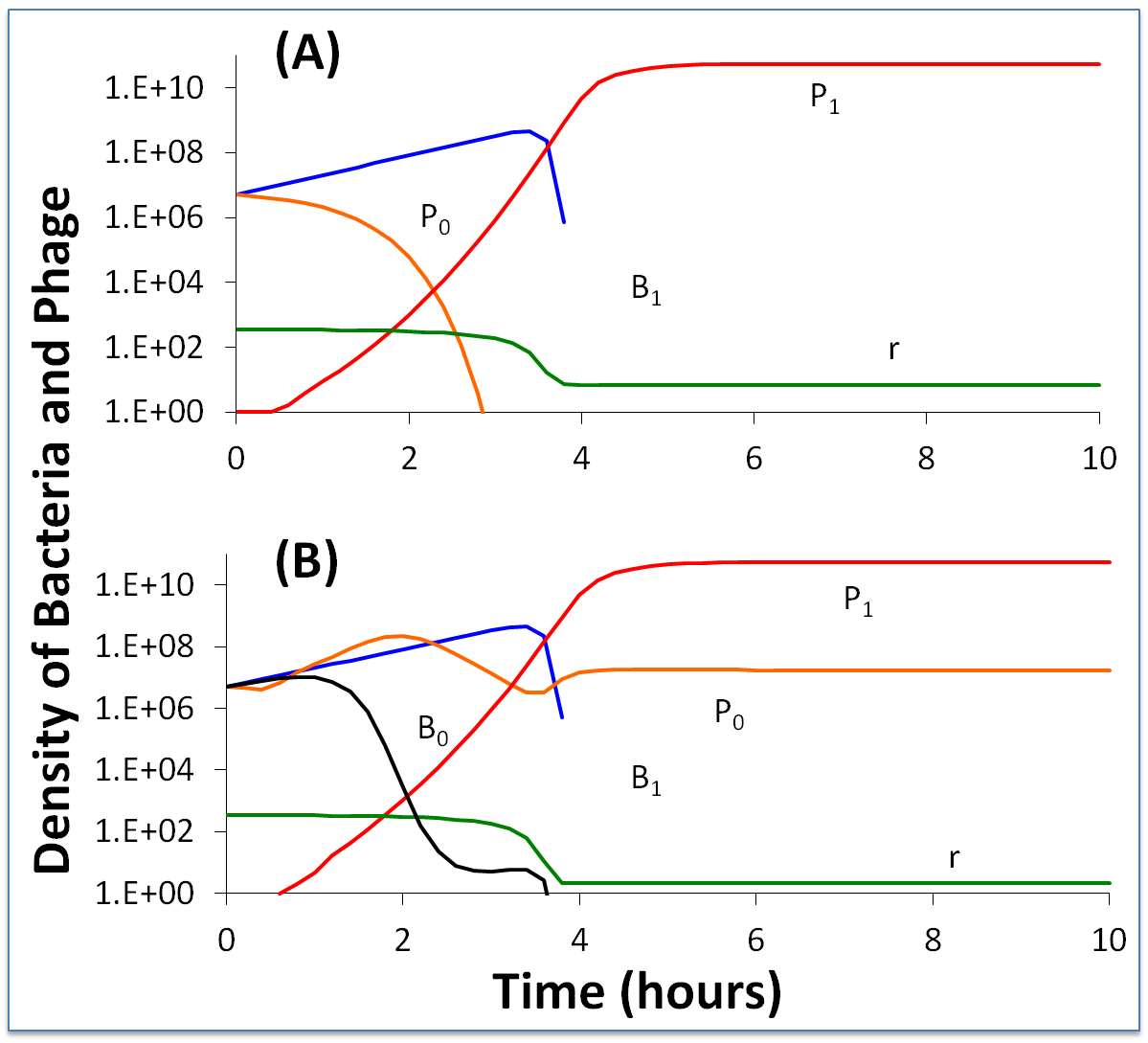

Supplement: Figure S3 — Simulation results showing changes in the density of bacteria and phage as well as the concentration of the limiting resource r. Panel A, initially a population of bacteria (B1) immune to the dominant population of phage (P0) and a single CEM mutant (P1) capable of replicating on B1. Panel B, initially equal densities of bacteria sensitive and immune to the P0 phage (B0 and B1) and no CEM phage (P1 = 0), but mutation to CEM at a rate μ = 10−6 per particle per burst. The population growth and phage infection parameters used for these simulations are those in the legend of Figure 2. (TIF) [file pgen.1003312.s003.tif]

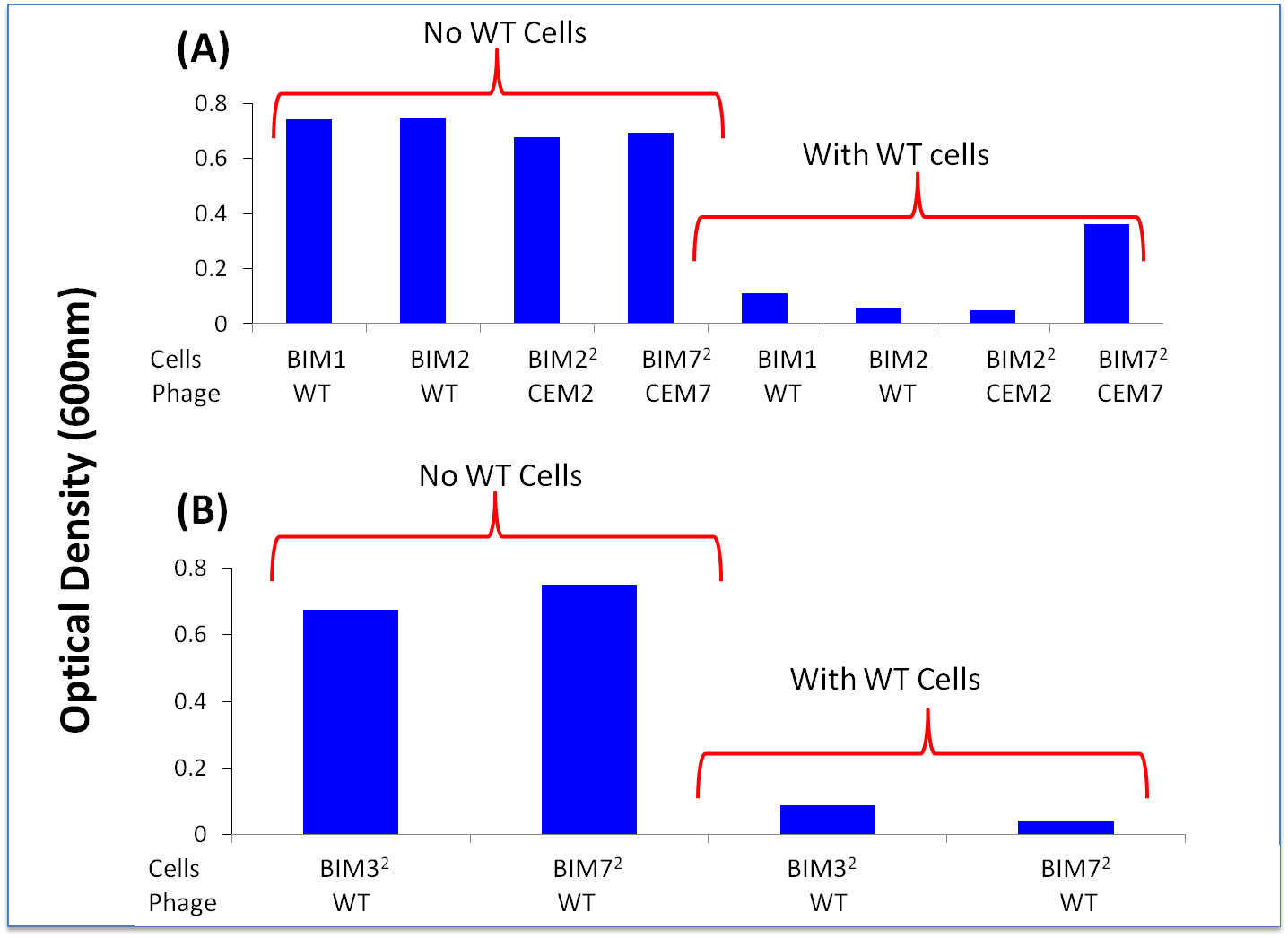

Supplement: Figure S4 — Optical density of cultures after 22 hours of incubation. Panel A, first-order BIMs (BIM1 and BIM2) with low densities (2×10^4^ pfu per ml) of WT phage or second-order BIMs (BIM32 and BIM72) with first-order CEMs (CEM3 and CEM7). Panel B, second-order BIMs with low densities (2×10^4^ pfu per ml) of WT phage with and without WT cells. (TIF) [file pgen.1003312.s004.tif]
