# Supplementary material for: The Population and Evolutionary Dynamics of Phage and Bacteria with CRISPR–Mediated Immunity
Source: PLoS Genet. 2013 Mar 14;9(3):e1003312. doi: 10.1371/journal.pgen.1003312 (PMC3597502; doi:10.1371/journal.pgen.1003312)
Supplement: Table S1 — List of new spacers acquired in CRISPR1 or CRISPR3 of S. thermophilus DGCC7710 and the corresponding protospacer regions in phage 2972. (PDF) [file pgen.1003312.s005.pdf]

Locus	Label	Novel Spacer Number	Protospacer location on WT Φ2972	Protospacer	Adjacent 7bp
CRISPR1	BIM1	1	5883..5912	TTTGAGTTTGAAAAACTCAACATGGCAGTT	AA**AGAA**G
	BIM3	1	30111..30082	AACATCGCCATGCATGTCCACGTCAATGAC	AA**AGAA**T
	BIM4	1	5720..5749	AACAGCCTTGAGTAATCATAAGAAGGGCGA	AG**AGAA**A
	BIM5	1	27380..27409	TCTGGAAAGCATATTGAGGGAGCTACTCTT	GA**AGAA**A
	BIM7	1	14289..14318	CAGCGCGGCCTCACAGGTGCAATCAGCAAT	GC**AGAA**T
	BIM11	1	29246..29275	GAAGTTGAAATAATTCGAGAAATAGAACTC	GA**AGAA**A
	BIM12	1	31582..31611	CTCAGTCGTTACTGGTGAACCAGTTTCAAT	TG**AGAA**A
	BIM3^{2}	2	23334..23363	AAGGAGCTAGCCACATTTCCGCAATTGATA	CT**AGAA**A
	BIM3^{2}	1	30111..30082	AACATCGCCATGCATGTCCACGTCAATGAC	AA**AGAA**T
	BIM5^{2}	2	33146..33175	TTAAGATTGTACACAGTGGAGATGGTTGAG	GT**GGAA**T
	BIM5^{2}	1	27380..27409	TCTGGAAAGCATATTGAGGGAGCTACTCTT	GA**AGAA**A
	BIM7^{2}	1	14289..14318	CAGCGCGGCCTCACAGGTGCAATCAGCAAT	GC**AGAA**T
CRISPR3	BIM2	1	25379..25408	CGTGCCAAGTCTGGTATAATAGTATCAGAA	A**GGTG**TA
	BIM6	1	18610..18581	TGAGTATGTATAGGACTTAACGAAAATCGT	T**GGAG**CA
	BIM8	1	30006..29977	CATTGGTGGTTTGTCAGCGAAAGAAATAAG	A**GGGGT**T
	BIM9	1	25848..25877	CATCACAGACACAGGAGAAGGTGGCTATTA	T**GGTG**GC
	BIM10	1	18374..18403	ACATCTGGAACAGTAGCACCAACGAATGGT	T**GGAG**TA
	BIM1^{2}	2	23182..23211	TCTGACGGTTAGATATAATTTTACTGGTAA	T**GGGG**AT
	BIM1^{2}	1	30006..29977	CATTGGTGGTTTGTCAGCGAAAGAAATAAG	A**GGGG**TT
	BIM2^{2}	2	1302..1273	GCTTCCTGCCTTGATGACCTCAGAGATGGA	C**GGGG**TT
	BIM2^{2}	1	25379..25408	CGTGCCAAGTCTGGTATAATAGTATCAGAA	A**GGTG**TA
	BIM4^{2}	1	30006..29977	CATTGGTGGTTTGTCAGCGAAAGAAATAAG	A**GGGG**TT
	BIM7^{2}	1	2343..2372	AGTCTGAACTTATGGGAAACCATAAGAAGC	A**GGGG**AT
	BIM8^{2}	2	34697..34704 1..22	CTTGTAGTGGTTCGAAAATTACATTAAGTT	T**GGAG**GT
	BIM8^{2}	1	30006..29977	CATTGGTGGTTTGTCAGCGAAAGAAATAAG	A**GGGG**TT
