# Supplementary material for: The Population and Evolutionary Dynamics of Phage and Bacteria with CRISPR–Mediated Immunity
Source: PLoS Genet. 2013 Mar 14;9(3):e1003312. doi: 10.1371/journal.pgen.1003312 (PMC3597502; doi:10.1371/journal.pgen.1003312)
Supplement: Table S2 — Mean and standard deviation in optical density (600 nm) with and without S. thermophilus SMQ-301. (DOCX) [file pgen.1003312.s006.docx]

|  | **Original** | **Original + SMQ-301** | **Diluted** | **Diluted + SMQ-301** |
| --- | --- | --- | --- | --- |
| **Mean** | 0.11 | 0.79 | 0.06 | 0.92 |
| **Std. Error** | 0.01 | 0.05 | 0.01 | 0.03 |
