# Supplementary material for: The Population and Evolutionary Dynamics of Phage and Bacteria with CRISPR–Mediated Immunity
Source: PLoS Genet. 2013 Mar 14;9(3):e1003312. doi: 10.1371/journal.pgen.1003312 (PMC3597502; doi:10.1371/journal.pgen.1003312)
Supplement: Text S1 — Evidence for Phage-independent inhibition of replication of BIMs. (DOC) [file pgen.1003312.s007.doc]

**Supporting Material Text S1**

**Evidence for Phage-independent inhibition of replication of BIMs.** The results of our experiments clearly indicate that a substance released from phage-lysed WT cells prevents the growth of BIMs. While there are an abundance of resources for these bacteria to grow in lysates with phage to which they are genetically resistant, they do not ascend. Not clear is whether this substance is a product phage infection, like a phag-encoded endolysin, or an inhibitory agent within viable cells. The results of the experiments described below support the hypothesis that this inhibiting substance can in part if not fully be attributed to something released by lysed *S. thermophilus* that does not require phage infection.

To test this hypothesis, we used a French Press - extracted lysate of a 38-hour stationary phase, phage-free culture of WT *S. thermophilus* DGCC7710*.* To produce this lysate, the culture was pressed twice at 180,000 PSI, centrifuged and passed through a 0.45 micron filter. The viable density of cells that passed through the French Press was 25% of the pre-pressed culture. To control for the possibility that BIMs (BIM2^2^) would not grow in this lysate derived from stationary phase cultures because it was denuded of nutrients, this FP lysate was mixed with fresh LM17Ca at ratios of 1:1, 1:2, and 1:5. In parallel we performed the same experiment with a centrifuged and filtered lysate prepared with phage WT phage 2972. To 3 ml each of the lysates and mixtures of lysate and medium we added 3 µl of a LM17Ca diluted overnight culture of BIM2^2^ cells for an initial density approximately 10^3^/ml and incubated them at 42^o^C.

If the French Press (FP) lysate was completely denuded of nutrients but contained nothing to inhibit the replication of the BIMs, after the cultures grew the ODs and viable cell densities in these 1:1, 1:2 and 1:5 lysate to medium cultures would be respectively 1/2, 2/3^rd^s, and 5/6ths that of parallel control, medium only cultures. In Figure S5 we plot the ratios of the ODs and CFUs estimates of viable at different times relative to average OD and viable cell density lysate-free controls and that expected if there was no inhibition. The results clearly indicate that both the French Press and phage lysates inhibit the growth of BIM2^2^. At this juncture we do not know what this substance is, but clearly plan to find out. It is, however, clear that the bacteria have to be lysed to release this agent. The supernatants of un-lysed stationary phase WT, *S. thermophilus* do not inhibit the replication of BIM2^2^ (data not shown).


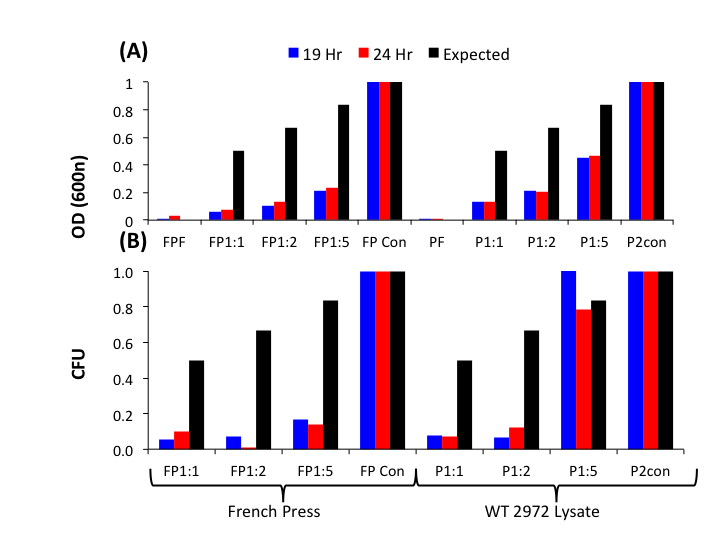


**Figure S5.** Relative growth of *S. thermophilus* BIM2^2^ in mixtures of fresh medium (LM17Ca) and French Press (FP) lysate of WT cells or WT phage 2972 (P) lysates. Panel A: Optical density of different mixtures of lysates and medium relative to the optical density of the lysate-free control (cells alone). FPF and PF are the ratios of the optical densities of the French Press and Phage lysates relative to the lysate-free control. Panel B: CFU estimates of viable cell densities of different mixtures of lysates and medium relative to the optical density of the lysate-free control. At the dilutions tested (10^-3^ and 10^-2^, respectively), there were no viable cells in the full-concentration French Press or phage lysates.
